# Fine-scale spatial variation shape fecal microbiome diversity and composition in black-tailed prairie dogs (*Cynomys ludovicianus*)

**DOI:** 10.1186/s12866-023-02778-0

**Published:** 2023-03-02

**Authors:** Sufia Akter Neha, Jorge Salazar-Bravo

**Affiliations:** 1 International Center for Arid and Semi-Arid Land Studies, Texas Tech University, Lubbock, TX 79409 USA; 2 Department of Biological Sciences, Texas Tech University, Lubbock, 79409 USA

**Keywords:** Geographic location, Host habitat, Environmental factors, *Cynomys ludovicianus*, 16S rRNA gene, Alpha diversity, Beta diversity

## Abstract

**Background:**

Host associated gut microbiota are important in understanding the coevolution of host-microbe, and how they may help wildlife populations to adapt to rapid environmental changes. Mammalian gut microbiota composition and diversity may be affected by a variety of factors including geographic variation, seasonal variation in diet, habitat disturbance, environmental conditions, age, and sex. However, there have been few studies that examined how ecological and environmental factors influence gut microbiota composition in animals' natural environments. In this study, we explore how host habitat, geographical location and environmental factors affect the fecal microbiota of *Cynomys ludovicianus* at a small spatial scale. We collected fecal samples from five geographically distinct locations in the Texas Panhandle classified as urban and rural areas and analyzed them using high throughput 16S rRNA gene amplicon sequencing.

**Results:**

The results showed that microbiota of these fecal samples was largely dominated by the phylum *Bacteroidetes*. Fecal microbiome diversity and composition differed significantly across sampling sites and habitats. Prairie dogs inhabiting urban areas showed reduced fecal diversity due to more homogenous environment and, likely, anthropogenic disturbance. Urban prairie dog colonies displayed greater phylogenetic variation among replicates than those in rural habitats. Differentially abundant analysis revealed that bacterial species pathogenic to humans and animals were highly abundant in urban areas which indicates that host health and fitness might be negatively affected. Random forest models identified *Alistipes shahii* as the important species driving the changes in fecal microbiome composition. Despite the effects of habitat and geographic location of host, we found a strong correlation with environmental factors and that- average maximum temperature was the best predictor of prairie dog fecal microbial diversity.

**Conclusions:**

Our findings suggest that reduction in alpha diversity in conjunction with greater dispersion in beta diversity could be indicative of declining host health in urban areas; this information may, in turn, help determine future conservation efforts. Moreover, several bacterial species pathogenic to humans and other animals were enriched in prairie dog colonies near urban areas, which may in turn adversely affect host phenotype and fitness.

**Supplementary Information:**

The online version contains supplementary material available at 10.1186/s12866-023-02778-0.

## Background

The host-associated microbiome influences potential host health and fitness and has spawned a growing interest in the scientific community. The microbiome study is important in understanding the coevolution of the host-microbe, its causes, and consequences in nature [1, 2]. Microbial communities of the mammalian gut are critical to digestive efficiency [3], behavior [4], homeostasis [5], nutrition [6], immune response [7], and pathogen invasion [8]. Shifts in microbial community composition have been associated with disruption and imbalanced gut microbiota that can lead to functional changes in the metagenome resulting in host morbidity and mortality [9]. Studies on the microbiome have typically been focused on humans and animals in laboratory settings, but there is still much to be learnt about gut microbial diversity and composition of hosts in a natural environment [4]. Fecal samples have been used as a proxy for reflecting microbial communities in the lower gastrointestinal tract of the host [10], however, the different regions of the gastrointestinal tract harbor different fermenters making it difficult to differentiate fecal bacteria from intestinal flora that may influence microbiome composition [11].

The gut microbiome offers a new perspective on the response of the host organism to geographic variation [12, 13] even with relatively small distances ca. 100 km between sampling sites or between countries [12, 14, 15]. For example, trapping location was found to account for 15% variation of the total gut microbiota in wild mice in Scotland [12]. The effect of geographic distance on microbiome composition was also documented in red squirrels among samples within a few kilometers of each other in Canada [4]. Biogeographic variation in gut microbiota is shaped largely by dispersal ability, and potentially other ecological factors affecting the local environment, food resources, and livestock farming intensity throughout sampling sites [15]. Geographic variation may also be related to differences in individual host factors that influence gut microbiome diversity, suggesting that bacteria colonizing the gastrointestinal tract are either promiscuous or acquired through environmental exposure [16].

Habitat is one of the key factors shaping gut microbiota in wild mammals and understanding the interaction between host habitat and microbial community structure could be useful for effective management plans [17, 18]. Animals living near urban areas show dramatically altered host physiology [19], movement patterns [20], foraging habits [21], pollution exposure [22], and vulnerability to predation [23]. Access to anthropogenic food resources in urban settings changes the gut microbiota composition in some rodents and carnivores, which leads to diet-induced obesity and hyperglycemia [24–26]. The effect of urbanization on bird populations has been demonstrated by substantial differences in microbial community structure and diversity as well as the taxonomic and functional composition of gut microbes [27, 28]. The relationship between gut microbiota characteristics and habitat type can be explained by various mechanisms. First, the gastrointestinal tract may be a repository for samples of the microbial communities present in the external environment such as soil and water resulting in local microbiome differences [29–31]. Second, the microbial community composition varies dramatically among hosts based on their genetic and phenotypic traits, suggesting that host filtering may favor specific bacterial communities [32]. Third, a diet-mediated shift that depends on both host-specific traits and host environments [27] has been documented in mammals and birds resulting from the provision of supplementary foods [33, 34].

Seasonal shifts may also play a critical role in shaping the structure of the microbial community. The gut microbiome of wild mammals changes rapidly with the seasons [35–37]. To illustrate, bacterial taxa involved in the production of amino acids and lipid metabolism were higher during dry seasons which may suggest that energy production and cellular activity may allow wild geladas (*Theropithecus gelada*) to switch their diet to starch in order to maintain energetic demands in periods of nutrient restriction [35]. In addition to seasonal and temporal variation, numerous studies have shown the association between spatial variation in environmental conditions such as temperature, precipitation, elevation, and gut microbiota composition. For example, temperature-driven microbiome variation in both fruit flies and humans has been reported across latitude [38, 39]. Another study found considerable changes in gut microbiota across an elevational gradient in a toad-headed lizard population [40]. As a result of seasonal and spatial variations in microbiome composition, certain bacteria may become more abundant which improves the metabolism of the host, while reducing the abundance of other microbes that affect host immunity [41, 42].

Black-tailed prairie dogs (*Cynomys ludovicianus*) are medium-sized rodents widely distributed in the North American Great Plains, from southern Canada to northern Mexico [43]. This species plays a key role in the short-grass prairie ecosystems of North America, providing prey and shelter for other species as well as contributing to soil texture and composition [43]. Because of their keystone function, this species is ideal to examine the association between gut microbiota diversity and composition in relation to spatial variation in host habitat and environment. Previous work in the fecal microbial diversity and abundance of the species only analyzed a small sample (*n* = 10) in the Janos Biosphere Reserve in Mexico [44]. Recent study examined the fecal and cecal microbiota of black-tailed prairie dogs in Kansas focused on 58 samples and compared sex and geographic variation between groups [45]. However, the response of fecal microbiota to habitat and environmental parameters has yet to be analyzed in detail.

The aims of the study were to (1) characterize the fecal microbiota of black-tailed prairie dogs in the Texas Panhandle; (2) determine how fecal microbiome diversity and composition vary with geographic location and habitat; and (3) test whether spatial variation in temperature, precipitation, and elevation affect fecal microbial diversity. Animals living in different eco-regions are exposed to different types of vegetation and diet, we hypothesize that the fecal microbiota of black-tailed prairie dogs occupying different geographic locations will develop different fecal microbiomes. Moreover, we predict that prairie dogs inhabiting urban areas closer to human settlements will exhibit reduced microbial diversity and richness than their counterparts in more rural areas. This is because urban habitats are characterized by reductions in plant species diversity resulting from biotic homogenization. In addition, we expect differential microbial abundance and significant changes in microbial community structure. Overall, we expect that ecological factors will be strongly related to prairie dog microbiome variation. To explore the relationship between host geographic location, habitat, environmental conditions, and fecal microbiome composition, the current study analyzed 70 fecal samples from 58 burrows in five geographically distinct colonies along a habitat gradient in the Texas Panhandle: urban areas (Dallam and Randall), and rural areas (Lubbock, Bailey, and Hockley). Analyses reveal new insights into the microbial structure and how they co-vary with predicted factors that offer new concerns for the conservation and management of the prairie dog population.

## Results

The raw sequences of 70 fecal samples totaled 5,407,660, with an average of 77,252 reads per sample (range 28,178 – 357,624; SD = 41,072). After quality filtering (Q > 30) and denoising, resulting on a grand total of 3,939,947 non-chimeric sequences, averaging 56,285 reads per sample (range 15,839 – 256,779; SD = 30,147). We detected a total number of 5,118 ZOTUs (zero-radius operational taxonomic units) by clustering all these reads based on a 100% similarity threshold. Observed OTUs leveled off at a sequencing depth of 40,000 indicated by the fact that enough OTUs have been detected to adequately characterize the microbial communities and the number of reads is not a limiting factor for OTU detection beyond 40,000 reads (Fig. S1). Some samples were characterized by a higher percentage of bacterial species than others and a shallow gradient from all samples indicated the relative abundance and incidence of those species were more evenly distributed. This observation was supported by the species rank abundance plot and the incidence abundance plot, as they showed a similar pattern across the dataset (Fig. S2A-B).

### Fecal microbiota composition

**Fig. 1:**
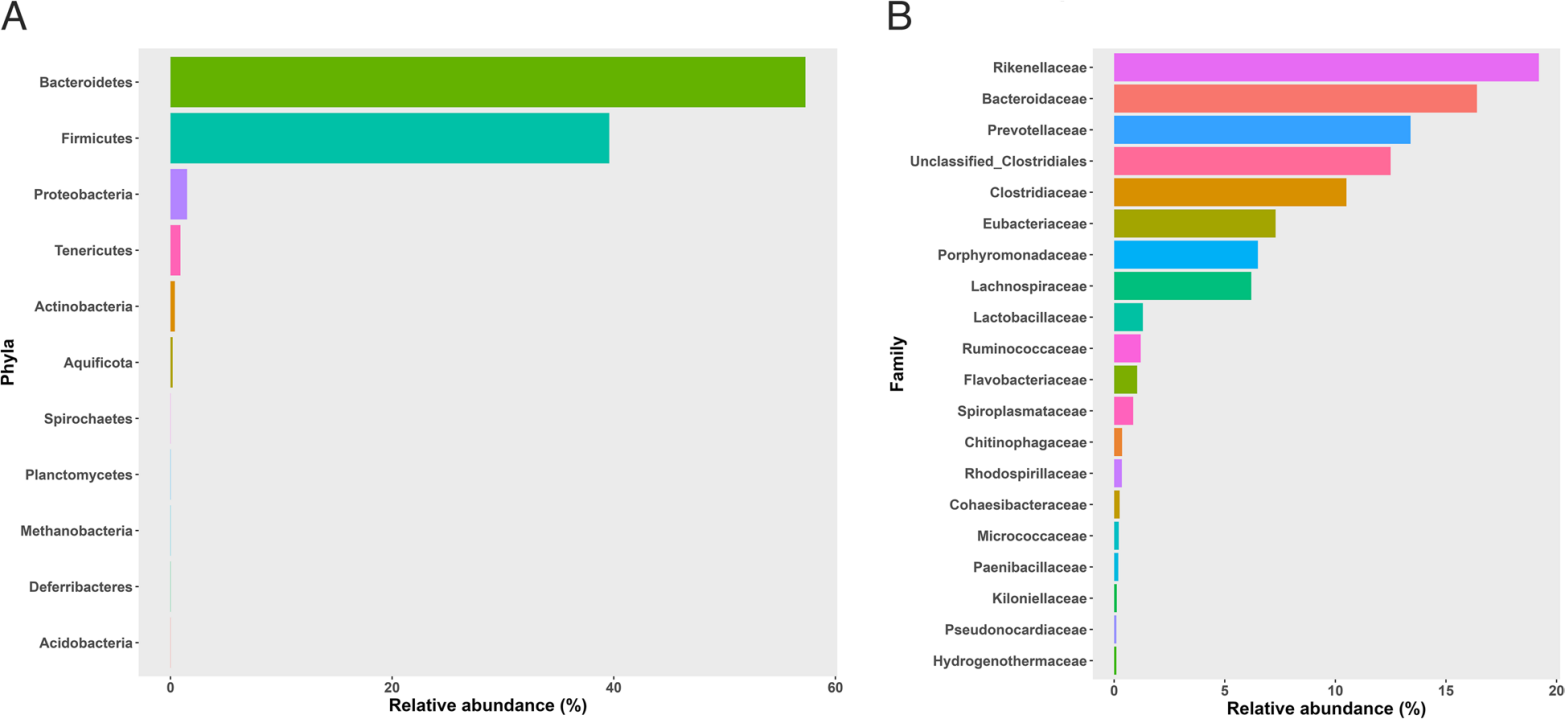
Relative abundance of bacterial taxa by phyla and family (**A**) eleven major phyla, and (**B**) top 20 bacterial families found in 70 fecal samples

Overall, 11 phyla, 57 families, 114 genera, and 220 species were found in all samples of *Cynomys ludovicianus*. *Bacteroidetes* was the most abundant phyla representing 57.3% of the fecal samples followed by *Firmicutes* (39.6%), *Proteobacteria* (1.5%), *Tenericutes* (0.9%), and *Actinobacteria* (0.4%) (Fig. 1A). The top ten families, *Rikenellaceae* (19.2%), *Bacteroidaceae* (16.7%), *Prevotellaceae* (13.4%), *Unclassified_Clostridiales* (12.5%), *Clostridiaceae* (10.6%), *Eubacteriaceae* (7.3%), *Porphyromonadaceae* (6.5%), *Lachnospiraceae* (6.3%), *Lactobacillaceae* (1.3%), and *Ruminococcaceae* (1.2%), made up 95% of the fecal microbiota (Fig. 1B). The most abundant genera dominated in all samples including *Alistipes* (19.2%), *Bacteroides* (16.5%), *Prevotella* (12.2%), *Eubacterium* (7.3%), *Clostridium* (6.4%), *Blautia* (5.8%), *Parabacteroides* (4.2%), *Anaerovorax* (3.8%), *Roseburia* (3.3%), and *Alkaliphilus* (1.7%). Microbial communities were dominant by five species, *Alistipes shahii* (13.1%), *Prevotella shahii* (10.4%), *Bacteroides rodentium* (7.3%), *Eubacterium oxidoreducens* (3.9%), and *Anaerovorax odorimutans* (3.8%) with the remaining species accounting for less than 5% of the mean relative abundance.

### Fecal microbiota alpha diversity

**Fig. 2:**
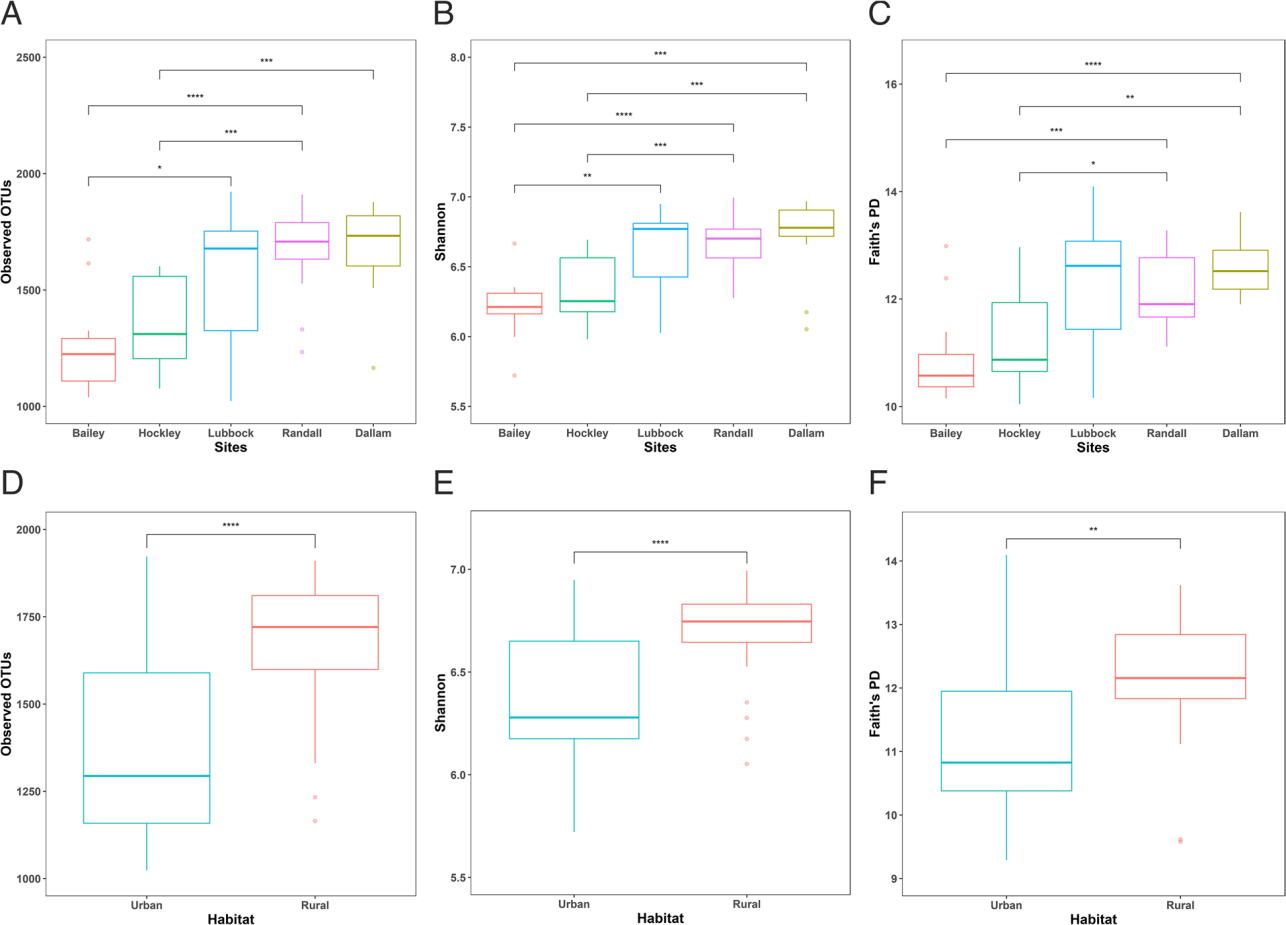
Box-and-whisker plots show alpha diversity comparisons of each group of prairie dogs' fecal microbiome at different sampling sites and habitats (**A**, **D**) Observed OTU richness, (**B**, **E**) Shannon index, and (**C**, **F**) Faith’s PD. Statistical significance ****p < 0.0001, ***p < 0.001, **p < 0.01, *p < 0.05 given by Tukey’s HSD test

The number of observed OTUs varied from 647 to 1922 per sample (Table S1). A strong linear relationship was found between the observed OTU richness and the total read counts of the sample in the dataset (Welch Two Sample t-test: t = 14.349, df = 69.077, *p* < 0.001). Microbial alpha diversity was summarized as observed OTU richness (R = 1474.3 ± 25.54), Shannon diversity index (H’ = 6.45 ± 0.04), as well as Faith’s phylogenetic diversity (PD = 11.55 ± 0.14) which was followed by the analysis of variance among sampling sites. There were no significant spatial autocorrelations in Moran's I test for alpha diversity metrics (Observed: *p* = 0.838; Shannon: p = 0.781; Faith’s PD: *p* = 0.522). The distribution of observed OTUs was significantly varied across sites (ANOVA, F = 9.90, *p* < 0.01; Fig. 2A, Table 1). Significant differences were present between alpha diversity estimates of five different experimental groups (Shannon: F = 5.27, *p* = 0.00097; Faith’s PD: F = 5.01, *p* = 0.0013; Fig. 2B and C). There was no statistical difference between Dallam and Randall regarding intra-individual diversity (Fig. 2A-C). For all three alpha diversity metrics, diversity and richness were significantly greater in Dallam, while lower in Bailey (Tukey’s HSD test, *p* < 0.05; Fig. 2A-C). We found that adding habitat as a covariate in the linear regression model increased the association between alpha diversity and sampling sites (8.0%), indicating part of the association was explained by habitat (*p* < 0.001; Table 2). Multiple linear regression analysis revealed a significant relationship between alpha diversity and the two predictor variables (F_(4,65)_ = 9.901, R^2^ = 0.38, *p* < 0.001). Significant variation was observed in alpha diversity between habitats (Observed: F = 31.38, *p* < 0.001; Shannon: F = 14.50, *p* = 0.0003; Faith’s PD: F = 12.45, *p* = 0.0007; Table 2). Diversity was significantly higher in rural areas compared to their counterparts in urban areas (Tukey’s HSD test, *p* < 0.001; Fig. 2D-F).

**Table 1:** Alpha diversity estimates in fecal microbiome of *Cynomys ludovicianus* across sampling sites and habitat

Sampling sites	Samples	Total reads	Reads after filtering	Observed OTU’s	Shannon	Faith’s PD
Lubbock	*n* = 12	69,949 ± 5780	51,434 ± 4548	1481.1 ± 116.8	6.49 ± 0.15	11.79 ± 0.462
Hockley	*n* = 14	72,097 ± 6392	51,945 ± 4690	1321.7 ± 54.2	6.33 ± 0.06	10.95 ± 0.295
Bailey	*n* = 16	92,082 ± 19,018	68,099 ± 13,872	1244.6 ± 47.5	6.21 ± 0.04	10.86 ± 0.197
Randall	*n* = 15	80,300 ± 6162	57,487 ± 4504	1678.1 ± 49.4	6.59 ± 0.09	11.91 ± 0.241
Dallam	*n* = 13	67,783 ± 5025	49,508 ± 3698	1670.0 ± 54.0	6.71 ± 0.08	12.37 ± 0.271
**Habitat**						
Rural	*n* = 28	74,488 ± 4147	53,783 ± 3007	1678.9 ± 35.7	6.64 ± 0.06	12.12 ± 0.182
Urban	*n* = 42	79,094 ± 7735	57,953 ± 5681	1337.8 ± 43.5	6.33 ± 0.05	11.16 ± 0.186

**Table 2:** Coefficients from the linear regression model of predictor variables affecting fecal microbial alpha diversity (observed richness) of black-tailed prairie dogs

Coefficient	Estimate (β)	SE	t-value	*p*-value
Intercept	1244.62	60.81	20.469	< 0.001
Dallam	435.30	90.82	4.793	< 0.001
Hockley	77.09	89.01	0.966	0.3896
Lubbock	236.46	92.88	2.546	0.0133
Randall	433.44	87.41	4.958	< 0.001
Intercept	1337.88	38.50	34.746	< 0.001
Rural	347.05	60.88	5.602	< 0.001

### Fecal microbiota beta diversity

**Fig. 3:**
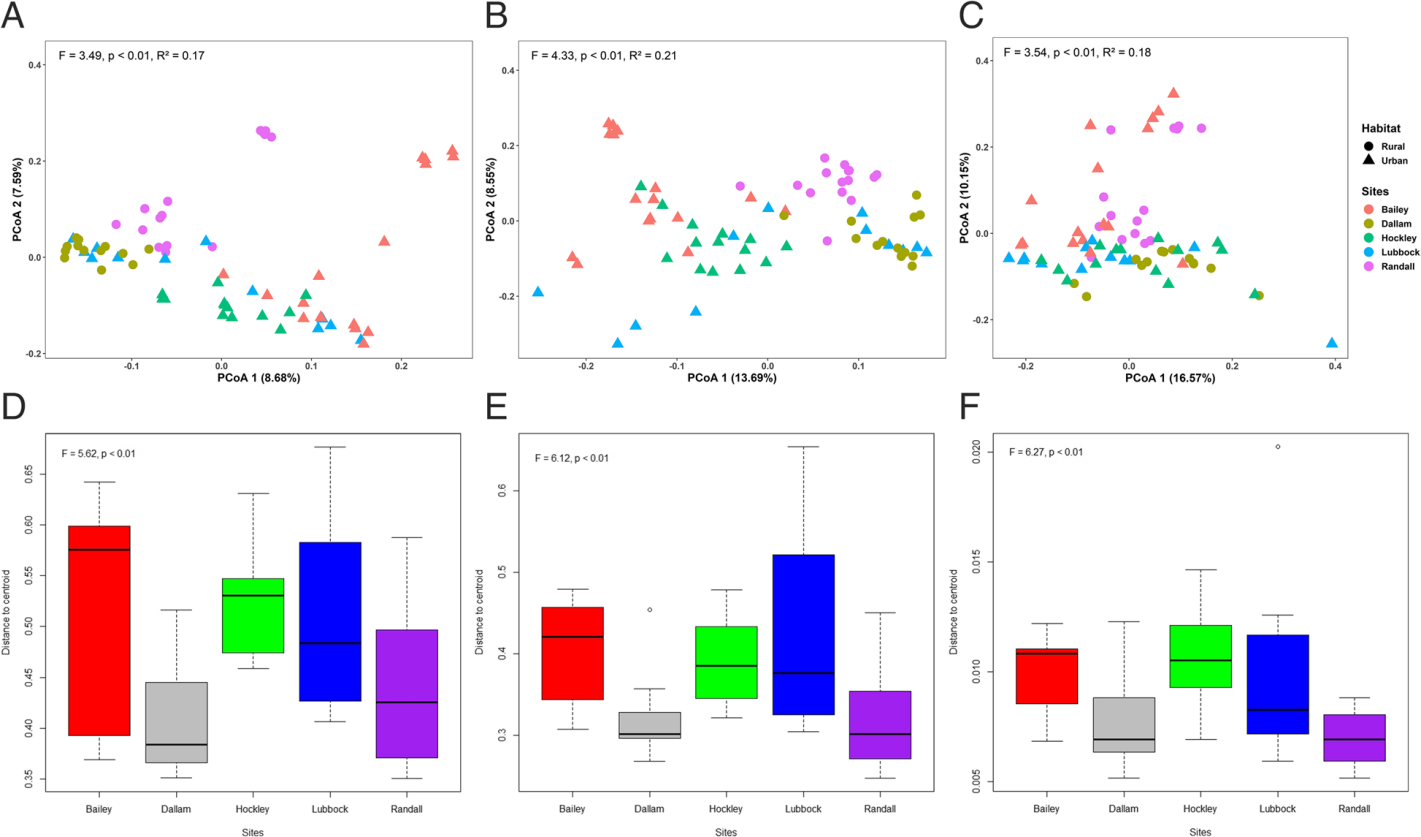
Beta diversity of microbial communities at five sampling sites estimated as: (**A**) Principal coordinate analysis (PCoA) based on Bray–Curtis dissimilarity index; (**B**) unweighted UniFrac distance, and (**C**) weighted UniFrac distance; colored by sampling sites and shaped by habitat and are for ADONIS test to study the effect of sampling sites on community matrix. Group dispersion based on (**D**) Bray–Curtis dissimilarity index, (**E**) unweighted UniFrac distance, and (**F**) weighted UniFrac distance; and are for ANOVA test for differences in dispersion from group centroid

The community membership as summarized by Bray–Curtis dissimilarities, unweighted and weighted UniFrac distances was significantly explained by sampling sites (F = 3.49, df = 4, *p* < 0.01; F = 4.33, df = 4, *p* < 0.01; F = 3.54, df = 4, *p* < 0.01, respectively) as well as habitat (F = 3.98, df = 1, *p* = 0.001; F = 5.74, df = 1, *p* = 0.001; F = 3.95, df = 1, *p* = 0.001, respectively) in a multiple predictor PERMANOVA model. This test was followed by post hoc pairwise testing between sites using permutational multivariate anlayses of variance using distance matrices (ADONIS) through which it was found that all comparisons were significant (*p* < 0.05; Table S2). The Mantel test showed that geographic separation of samples was correlated with community dissimilarity matrices (Bray–Curtis dissimilarities: *r* = 0.044, *p* = 0.003; unweighted UniFrac: *r* = 0.065, *p* = 0.024; weighted UniFrac: *r* = 0.013, *p* = 0.029). To evaluate the effect of sampling sites and habitat, principal coordinate analysis (PCoA) based on Bray–Curtis dissimilarities, unweighted and weighted UniFrac distances resulted in 16.27%, 22.24%, and 26.72% of the total variation in the community matrix being summarized on the first two axes (Fig. 3A-C). Multivariate homogeneity of group dispersions (β-dispersion) using Bray–Curtis, unweighted and weighted UniFrac distances corroborated significant variation in the dispersion of samples from group centroids across sampling sites (ANOVA: F = 5.62, *p* < 0.01; F = 6.12, *p* < 0.01; F = 6.27, *p* < 0.01, respectively; Fig. 3D-F).

### Relative abundance of bacterial taxa

**Fig. 4:**
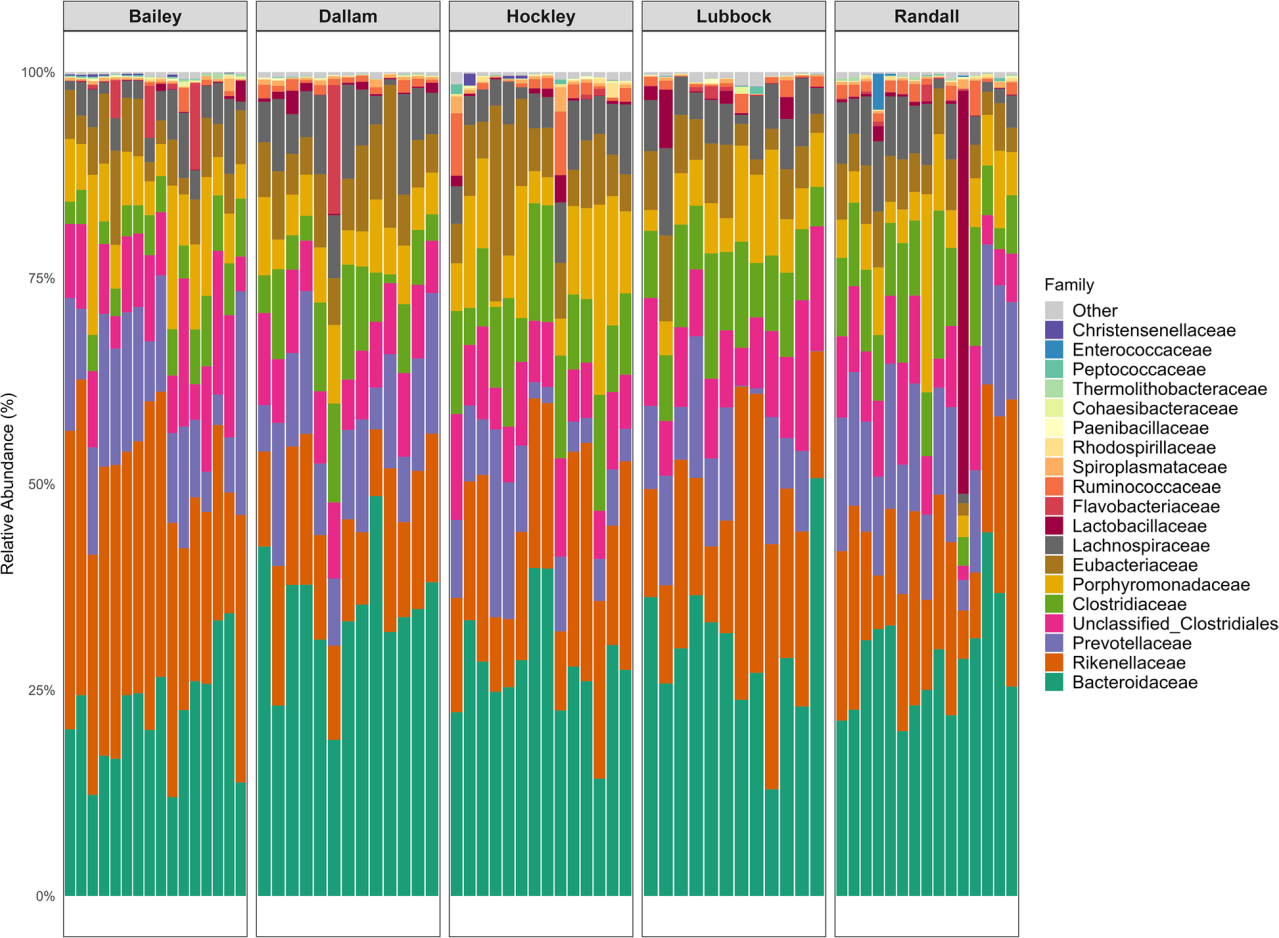
Relative abundance of bacterial communities where taxa represent the most abundant families for 70 fecal samples

**Fig. 5:**
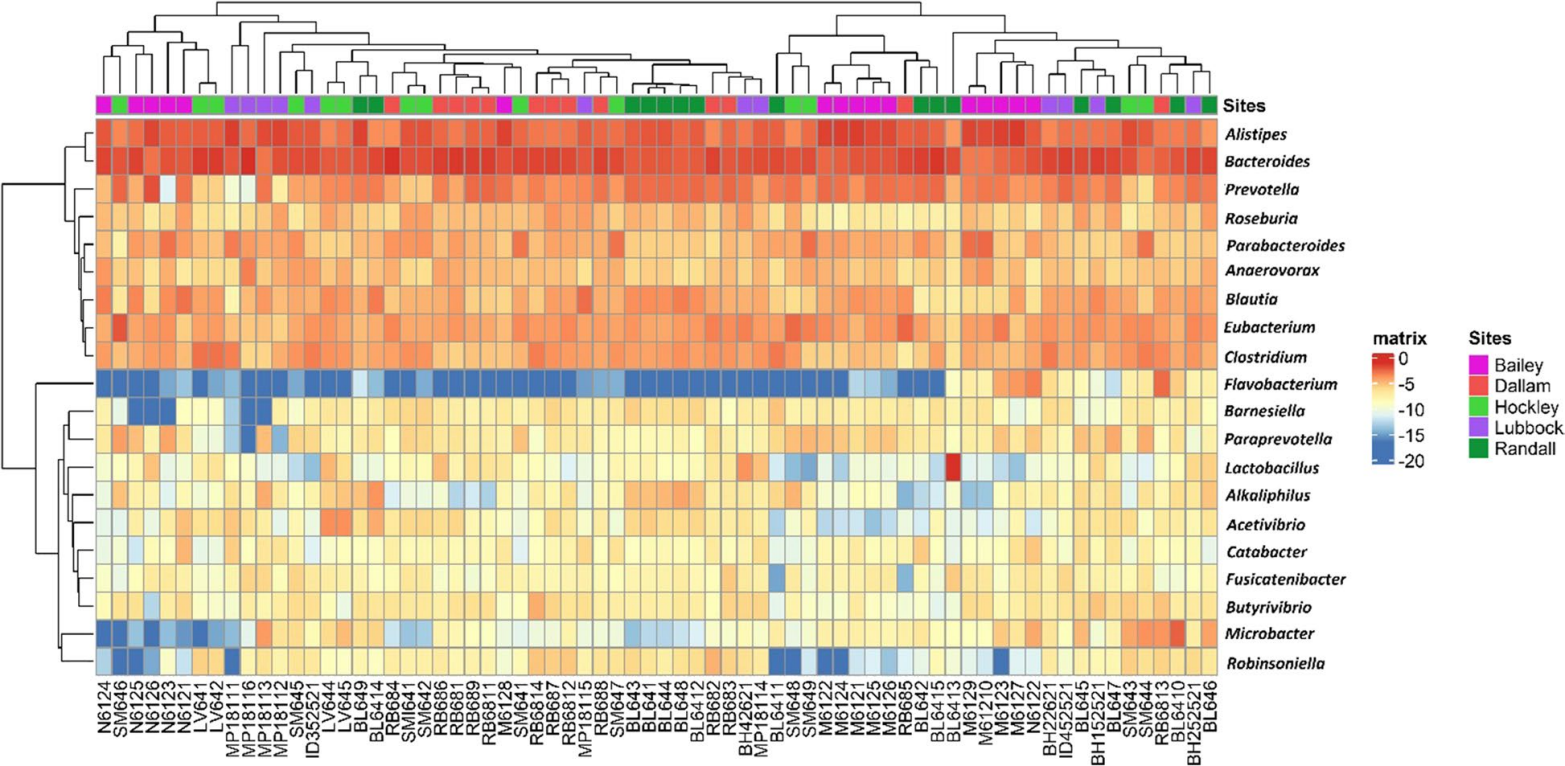
Heatmap showing hierarchical clustering of microbiome composition based on 16S rRNA amplicons for fecal samples. The microbiota shown represents the top 20 bacterial genera across all samples with the greatest mean relative abundance. The color of the heatmap of each taxon's relative abundances (from blue to red) is based on row-scaled data. Top dendrogram shows the samples with similar microbiomes clustered together whereas the side dendrogram portrays the bacteria tend to co-occur are clustered

To determine which taxa were contributing to the observed differences across sampling sites, we examined differences in relative abundance at the family level. Differences were present in the relative abundance of specific bacterial families: the most abundant family in Dallam being *Bacteroidaceae* (22.87%). *Prevotellaceae* (21.91%) was significantly higher in all samples in Randall County compared to other sites (Fig. 4). Bailey and Hockley were largely dominated by *Rikenellaceae* and *Porphyromonadaceae* (26.63% and 15.84% respectively), while Lubbock was mostly abundant with *Bacteroidaceae* (16.28%). A hierarchical clustering method based on the relative abundance of genera clearly separated the samples from each sampling site (Fig. 5). Two distinct bacterial groups were differentiated by their divergent abundance patterns in the samples. The highly abundant group in the samples included the genera *Alistipes, Bacteroides, Prevotella, Roseburia, Parabacteroides, Anaerovorax, Blautia, Eubacterium*, and *Clostridium*. On the other hand, the group displayed the contrasting patterns constituting *Flavobacterium, Barnesiella, Paraprevotella, Lactobacillus, Alkaliphilus, Acetivibrio, Catabacter, Fusicatenibacter, Butyrivibrio, Microbacter*, and *Robinsoniella*. The latter group, however, included genera (e.g., *Alkaliphilus*, *Fusicatenibacter, Robinsoniella*) causing infection in humans [46–48]. Moreover, Bailey showed higher contributions of *Alistipes* (18.37%) whereas *Parabacteroides* (9.15%) mostly prevailed in Hockley. Both Dallam and Lubbock had a greater abundance of *Bacteroides* (27.74%, 22.21%, respectively). Prairie dogs from Randall County had higher relative abundance of *Prevotella* (9.38%).

### Differential abundance testing

**Fig. 6:**
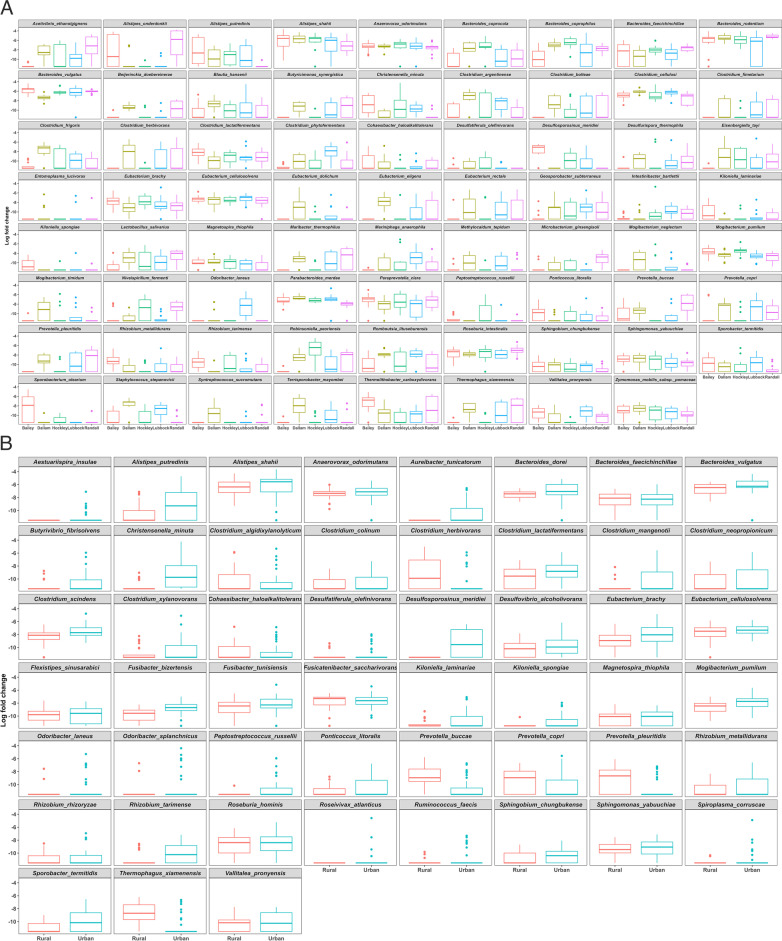
Differential abundance analysis of the bacterial taxa in the fecal microbiome among (**A**) sampling sites and (**B**) habitats based on log fold change data derived from ANCOMBC. Differential abundance testing was performed for 220 bacterial species in the dataset using DA methods. Each box represents one bacterial species found to be differentially abundant across the expermental groups and colored boxplots appear inside each box to show the groups being compared. Species were considered differentially abundant if their adjusted *p*-values < 0.05

Species-level comparisons were accounted for by differential abundance analyses (ANCOMBC) with 71 species observed to differ across five experimental groups (Fig. 6A). To provide a robust understanding of the microbiome differences between prairie dogs living in rural areas and those living in urban areas, we also used ANCOMBC. This analysis identified an apparent microbiome divide between prairie dogs in urban and rural settings and found 51 species that were differentially abundant between rural and urban habitats. A greater percentage of *Clostridium* sp., *Cohaesibacter haloalkalitolerans*, *Prevotella* sp., and *Thermophagus xiamenensis* were found in rural areas (Fig. 6B). In contrast, the fecal microbiota of urban areas were characterized by higher proportion of *Alistipes* sp., *Anaerovorax odorimutans*, *Aureibacter tunicatorum*, *Bacteroides* sp., *Butyrivibrio fibrisolvens, Christensenella minuta*, *Clostridium* sp., *Desulfosporosinus* sp.*, Eubacterium brachy, Fusibacter* sp*.*, *Kiloniella* sp., *Mogibacterium pumilum, Peptostreptococcus russelli, Ponticoccus litoralis, Rhizobium* sp., *Sphingobium* sp., *Sporobacter termitidis, and Vallitalea pronyensis*.

Furthermore, to explore the bacterial species contributing to the important values in the habitat, we used random forest classifier. We found *Alistipes shahii* had higher mean decrease accuracy (MDA = 3.53; zotu 745, 905, 906, 1041, 1401, 3705, and 4211) compared to others and thus had a greater impact on the accuracy of the classification followed by *Bacteroides rodentium* (MDA = 3.34; zotu 98, 589, 1351, 1572, 2937, 3210, 3354, 3787, 4298, and 4897) and *Prevotella shahii* (MDA = 2.83; zotu 386, 1847, 1893, 2113, 2777, and 2852) (Fig. S3).

### Relationship among environmental variables, fecal microbial alpha, and beta diversity

**Fig. 7:**
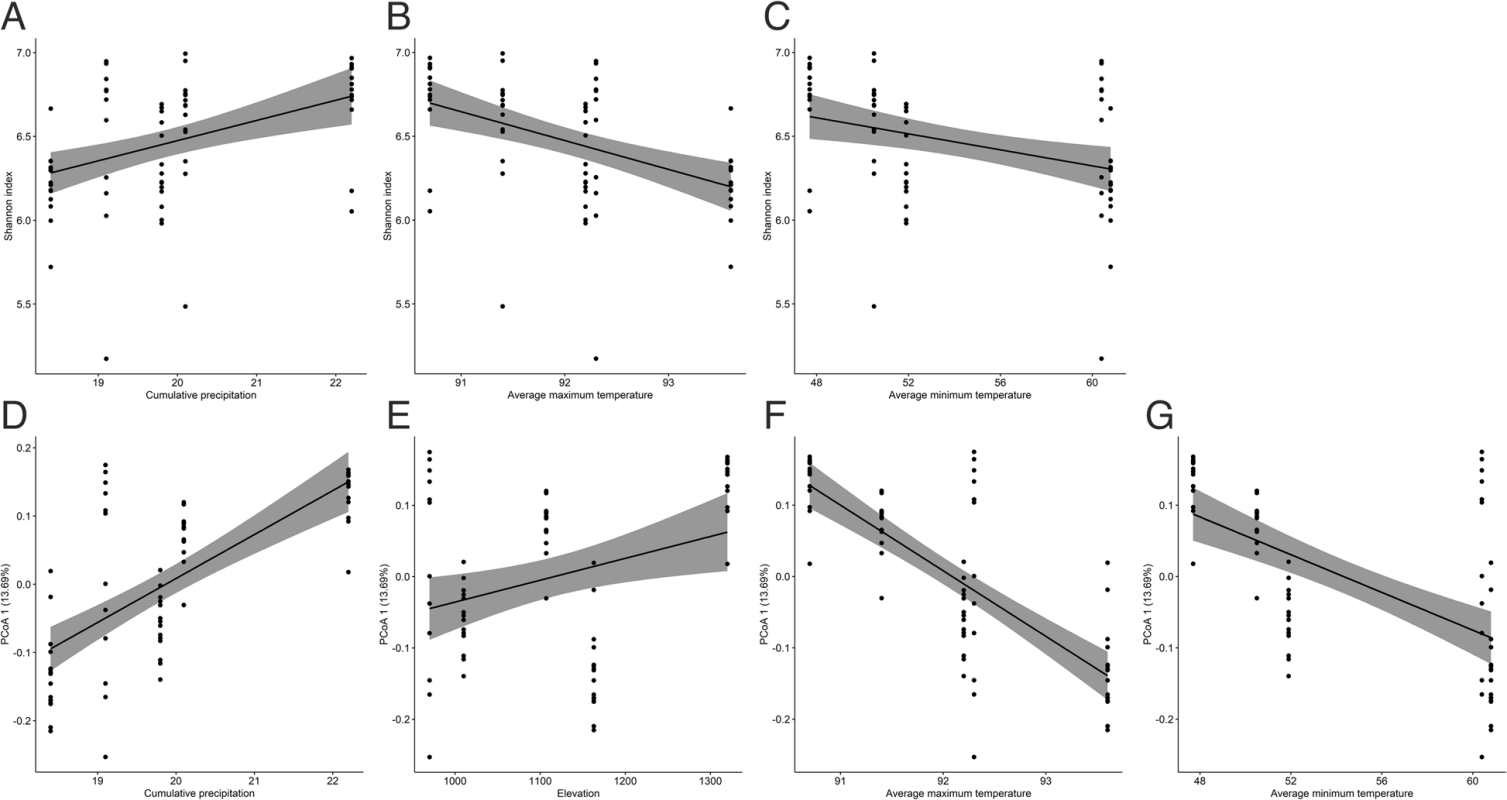
Environmental variables structure prairie dog fecal microbiome. The upper panel shows scatter plots of alpha diversity (Shannon diversity index) where the black line shows smoothed confidence intervals derived from linear regression (**A**) cumulative precipitation, (**B**) average maximum temperature, and (**C**) average minimum temperature. The lower panel demonstrates scatter plots of beta diversity on the first principal component (based on unweighted UniFrac distance) according to (**D**) cumulative precipitation, (**E**) elevation, (**F**) average maximum temperature, and (**G**) average minimum temperature

To investigate whether environmental factors were associated with the changes in black-tailed prairie dog fecal microbial diversity, we used linear and linear mixed effect models based on AICc. We observed a significant positive association between Shannon diversity index and cumulative precipitation (Pearson correlation: *r* = 0.42, *p* = 0.0003; Fig. 7A), but negative correlation with average maximum (*r* = -0.47, *p* < 0.001) and average minimum temperature (*r* = -0.35, *p* = 0.0033) (Fig. 7B and C). No significant correlation was observed between elevation and Shannon index (*r* = 0.19, *p* = 0.11). First principal component of PCoA (based on unweighted UniFrac distance) that explained 13.69% variation of the beta diversity was positively correlated with cumulative precipitation (*r* = 0.69, *p* < 0.001) and elevation (*r* = 0.31, *p* = 0.01) (Fig. 7D and E). In contrast, significant negative correlation of beta diversity was observed with both average maximum and average minimum temperature (*r* = -0.77, *p* < 0.001; *r* = -0.59, *p* < 0.001, respectively, Fig. 7F and G). In both alpha and beta diversity models, average maximum temperature was the strongest predictor considered the top ranked model (F_(1,68)_ = 18.97, R^2^ = 0.22, *p* < 0.001; F_(1,68)_ = 54.71, R^2^ = 0.39, *p* < 0.001, respectively; Table S3).

## Discussion

In summary, this study identified general patterns of the spatial variation in fecal microbiome diversity and composition in black-tailed prairie dog population in the Texas Panhandle. Our results demonstrate that diversity and composition of the microbiome vary strongly as a function of landscape and environment. Following were the key findings from this study: (i) the dominant phylum was *Bacteroidetes*, followed by *Rikenellaceae* as the dominant family, *Alistipes* as the dominant genus, and *A. shahii* as the dominant species; (ii) significant differences were observed in alpha diversity estimates across sampling sites and habitats; (iii) host geographic location and habitat were the strong predictors affecting fecal microbiome diversity and community composition; (iv) microbiome composition was affected significantly by differentially abundant bacterial taxa ; and (v) average maximum temperature explained fecal microbial alpha and beta diversity. Combined, these findings point to the ecological factors driving spatial patterns of the fecal microbiota of prairie dogs in their natural environment. Furthermore, our data suggest that shifts in microbiota composition and reduction in gut microbiota diversity in urban populations may negatively impact prairie dog health and fitness.

### Fecal microbiota composition

Overall, *Bacteroidetes* was the most dominant phylum in this study, with *Rikenellaceae* representing the most dominant group within it. These results are similar to studies on other rodents [49, 50] but not consistent with studies on the fecal and caecal microbiota of black-tailed prairie dogs in Kansas, USA [45] and fecal microbiota of *Cynomys ludovicianus* in Chihuahua, Mexico [44]. In particular, the proportion of *Bacteroidetes* accounted for less than 10% of the microbiota in previous studies of black-tailed prairie dogs (Table S4), versus 57.3% in this study. At the same time, *Firmicutes* abundance was lower in our study (39.6%) resulting in an overall increase in *Bacteroidetes* (Table S4). An increased relative abundance of *Bacteroidetes* with concomitant reduction of *Firmicutes* stimulates the host immune response resulting in either diet-induced obesity or weight loss due to a high-fat or high-fiber diet [51–55]. The phylum *Firmicutes* plays a dominant role in producing a health-promoting molecule in mammals called butyrate, and its abundance has been found to decrease significantly in comparison to other phyla in case of metabolic disorders in animals [9]. Several other studies suggest that a change in relative abundance with higher *Bacteroidetes* is associated with leanness, resulting in a decrease in *Firmicutes*/*Bacteroidetes* ratio [56–58]. Changes in abundance of *Bacteroidetes* and *Firmicutes* may be affected by geographical location, environment, and dietary pattern of the host. The variation in bacterial phyla may also be obtained in our study due to methodological differences in sampling techniques, DNA extraction protocols, primer design, and the variable regions being amplified. Within the phylum *Bacteroidetes*, the most abundant family was *Rikenellaceae* (19.2%) which may be associated with primary and secondary degradation of carbohydrates as suggested by previous studies [59]. The most abundant genus *Alistipes* (19.2%), and species *A. shahii* (13.1%) accompanied by a decrease in *Lactobacillus* (1.3%) in the present study was in agreement with Maurice et al. 2016, indicating an influence of seasonal differences in dietary intake on the microbial communities in the gut of wild wood mice. In the current study, samples were collected in the summer from early June to late August, thus future studies involving collecting samples year-round may reveal the seasonal dietary shifts in the black-tailed prairie dog fecal microbiota. Other studies have shown that high levels of *Alistipes* were observed in frail and aged populations of mice and humans [60, 61]. Since microbial communities have distinct functional patterns, examining functional metagenomic profiling, as well as their role in metabolic pathways, needs to be further explored.

### Reduced fecal microbial alpha diversity in urban populations

We found a significant relationship between the alpha diversity of prairie dog fecal microbiota and the geographic location of the prairie dog colonies sampled. Prairie dogs from five experimental groups showed nearly all significant differences in proportions in the analysis of variance which was later corroborated by the Tukey test. Dallam showed more diverse communities in three alpha diversity metrics. In contrast, Bailey—the more urbanized County—showed decreased richness and diversity. Different ecoregions were distinguished by the geographic locations in our study, such as riparian, rangelands, and high plains, which supported different topography and vegetation. Researchers have found that different fiber sources and proportions of fiber in the diet may contribute to regional gut microbiome differences [9, 62]; however, it is beyond the scope of this study to assess prairie dog diets at distinct geographical locations and how differences in feeding patterns correlate with fecal microbiome diversity. The alpha diversity of the fecal microbiome was influenced by the habitat. Prairie dogs living in rural habitats were found to maintain a diverse fecal microbe, whereas those living in urban habitats had reduced diversity due to a more homogenous environment, reduced foraging availability, lower coverage of vegetation, and anthropogenic disturbance (contact with domestic animals and humans, and their pathogens). In this scenario, rural ecosystems may be advantageous for this species. The findings are in line with those from other studies on birds, which revealed that altering diets in urban habitats can reduce alpha diversity owing to low-quality diets and access to novel anthropogenic food items [27, 63], thus leading to dysbiosis [64]. Impacts of urbanization on microbial diversity and functional composition of the fecal microbiota are appropriate next steps towards determining whether low diversity is related to lower hosts’ health.

### Microbial beta diversity is affected by both geographic location and habitat

We found that fecal microbial beta-diversity is shaped by geographic location and habitat in the study group. Prairie dogs from rural habitats tended to cluster together in an ordination plot more often than individuals from urban habitats. These taxonomic and phylogenetic patterns indicate the microbiomes are subjected to selective pressures on a fine spatial scale. Similar results have been observed for wild mice, in which the gut microbiota compositional differences were significantly affected by sampling distances as few as 1.5 km to 100 km [12, 14]. Based on the comparison of beta diversity across sites, unweighted UniFrac distances had greater explanatory power than weighted UniFrac distances, indicating that differences in beta diversity were more influenced by the presence or absence of microbial communities than by the relative abundances of taxa. ADONIS results showed that inter-individual variation in community composition across host populations and the significance of differences were supported in a pairwise fashion comparing between groups. Beta dispersion confirmed the variation in community structure and suggests shifting microbial community composition at a population level rather than an individual response to habitat and geographic location. A shift and increased dispersion or greater heterogeneity of variance in the community composition in urban areas indicating more changes in community structure due to selective pressure from human disturbance. More detailed data on the genetic diversity of the host population, food availability, and disease susceptibility will help us to detect variations in microbiota composition in urban prairie dog populations.

### Effect of urbanization on microbial community composition

Relative abundance analysis indicated that microbial community structure was influenced by geographic location. The higher relative abundance of *Bacteroidaceae* and *Prevotellaceae* in Dallam and Randall Counties in the feces of *C. ludovicianus* was mainly caused by the increased proportion of the genus *Bacteroide*s and *Prevotellla*. Fermenters belonging to the *Bacteroidaceae* and *Prevotellaceae* families have been found to degrade carbohydrates and breakdown of non-cellulosic polysaccharides related to an increase in fiber-rich diets [65–70]. These results imply that prairie dogs living in the rural areas might have abundant food supplies allowing them to obtain nutrients from grasses and other plant polysaccharides. In contrast, the prevalence of *Alistipes* from the *Rikenellaceae* family in Bailey may reflect an increase in bile acids triggered by dietary fat consumption, as demonstrated by human diet intervention study [71]. Hockley was dominated by members of the *Porphyromonadaceae* family, including *Parabacteroides*, a succinate-producing bacterium that may be linked to the consumption of flavonoid-rich foods. Recent studies have proposed the association between the intake of flavonoid-rich food items and the increased abundance of *Parabacteroides* in the gastrointestinal tract of humans [72, 73]. The population from these counties were closer to human settlements and subjected to consumption of human-mediated food resources including nuts, bread, berries, apples, meat products, and so on. While it is not clear whether dietary change alters the microbiome community structure, investigating the changes in gut microbiota associated with the degree of urbanization could shed light on the mechanisms behind these changes which potentially help wildlife managers to maintain and protect prairie dog populations.

### Response of host species to urbanization

Results of ANCOMBC tests showed that Randall and Dallam counties had higher levels of butyrate-producing bacteria (*Acetivibrio, Lactobacillus, Butyricimonas, Prevotella, Clostridia,* and *Eubacterium*). The production of butyrate is thought to promote health by supplying energy to intestinal epithelial cells in mice and rats [74–76]. In addition to butyrate-producing bacteria, Lubbock, Hockley, and Bailey also contained differentially abundant bacteria which were identified as pathogenic to humans and animals (*Intestinibacter bartlettii, Romboutsia* sp., and *Robinsoniella peoriensis*) [48, 77, 78]. Recent studies found *Odoribacter* sp. in the fragmented landscape with anthropogenic disturbance [79]. These areas also presented some newly designated bacterial species (*Sporobacterium olearium*, *Niveispirillum* *fermenti*); at this point it is unknown if host phylogeny and/or host physiological responses to diet are associated with the presence of these taxa [80]. Furthermore, the prevalence of *Bacteroides* and *Alistipes* in urban settlements suggested prairie dogs in these areas consumed high fat and protein diets which could contribute to metabolic disease risk [81, 82]. Alternatively, *Prevotella* is found in diets rich in fiber and is regarded as a marker of a healthy gut microbiome with low disease risk profiles [81, 82] has been found dominant in rural areas. Moreover, more differentially abundant bacteria in urban areas suggested the potential for adaptation of gut microbes leading to the conclusion of increased metagenome plasticity in prairie dogs due to selective pressures from anthropogenic disturbances as well as the loss of some species and the gain of others [79].

Our RF model identified the top 7 bacterial species (*Alistipes shahii, Bacteroides rodentium, Prevotella shahii, B. acidifaciens, B. stercorirosoris, B. dorei,* and *Roseburia Faecis*) among the model’s top 30 predictor variables. We detected the three most important species were *A. shahii, B. rodentium* and *P. shahii* which drove the changes in fecal microbiome composition. These groups of bacterial taxa have been considered core gut microbiota in healthy mice and humans [83].

### Environmental variables explain fecal microbial alpha and beta diversity

In addition to spatial structure contributing to microbiome variation, environmental variation may also influence microbiome differences in wildlife population mediated by host population genetics through the modulation of host factors including behavior, immunity, and physiology [84]. We found a strong influence of precipitation on prairie dog gut microbiome diversity and composition. The number of cellulolytic and fibrolytic bacterial taxa (*Bacteroidaceae* and *Prevotellaceae*) was higher during summer seasons when grass rich in cellulose was widely available. This pattern was also witnessed in other mammals [35, 37]. The gut microbial alpha diversity did not change along with a small variation in altitudinal gradient, but it partially influenced microbial beta diversity. Our results agreed with previous studies on other wild species, such as house mice (*Mus musculus domesticus*) [85], lizard (*Phrynocephalus vlangalii*) [40], macaque (*Macaca thibetana*) [86], and wild sable (*Martes zibellina*) [87], which indicatde that elevational gradient had an impact on gut microbial community composition. Several climatic conditions would explain this relationship, including oxygen concentration, ambient temperature, air pressure, and the composition of vegetation [85]. Both average maximum and average minimum temperatures were negatively associated with microbial alpha and beta diversity. Moreover, the average maximum temperature was the strongest predictor, explaining 39% of the overall microbiome community composition. In particular, during periods of high temperature, changes in the relative abundance of certain bacteria (increase in *Bacteroidetes* and decrease in *Firmicutes*) was observed in this study. It has been shown that each host displays its unique microbial response to heat stress, but there are some gut bacterial taxa, including lineages of *Firmicutes* and *Proteobacteria*, that seem to show consistent response to temperature variation across host species [88]. For example, the relative abundance of *Firmicutes* within the fecal microbiota of laying hens subjected to heat stress had been shown to decrease significantly [89]. The higher temperature was also associated with a decrease in overall alpha diversity in lizards [90]. Changing the composition of gut microbes may affect gene function, resulting in altered host phenotypes and fitness. Future studies should explore the association between temperature-induced changes and the composition of gut microbiota in prairie dogs and how they contribute to host phenotypic plasticity and fitness.

## Conclusion

Overall, our findings revealed that spatial variation in host geographic location, habitat and environmental factors affected the fecal microbiota of black-tailed prairie dogs. Reduction in alpha diversity in conjunction with greater dispersion in beta diversity could be indicative of declining host health in urban areas [91, 92]. Moreover, several bacterial species pathogenic to humans and other animals were detected with high abundance in urban areas indicating that host fitness might be negatively affected. A shift in gut microbial community composition has been related to numerous diseases and infections and could potentially promote co-infections [93–95]. Future research that incorporates a multi-omics approach including transcriptomics, genomics, proteomics, and metabolomics will uncover the metagenome plasticity and host fitness.

## Methods and materials

### Fecal sampling

**Fig. 8:**
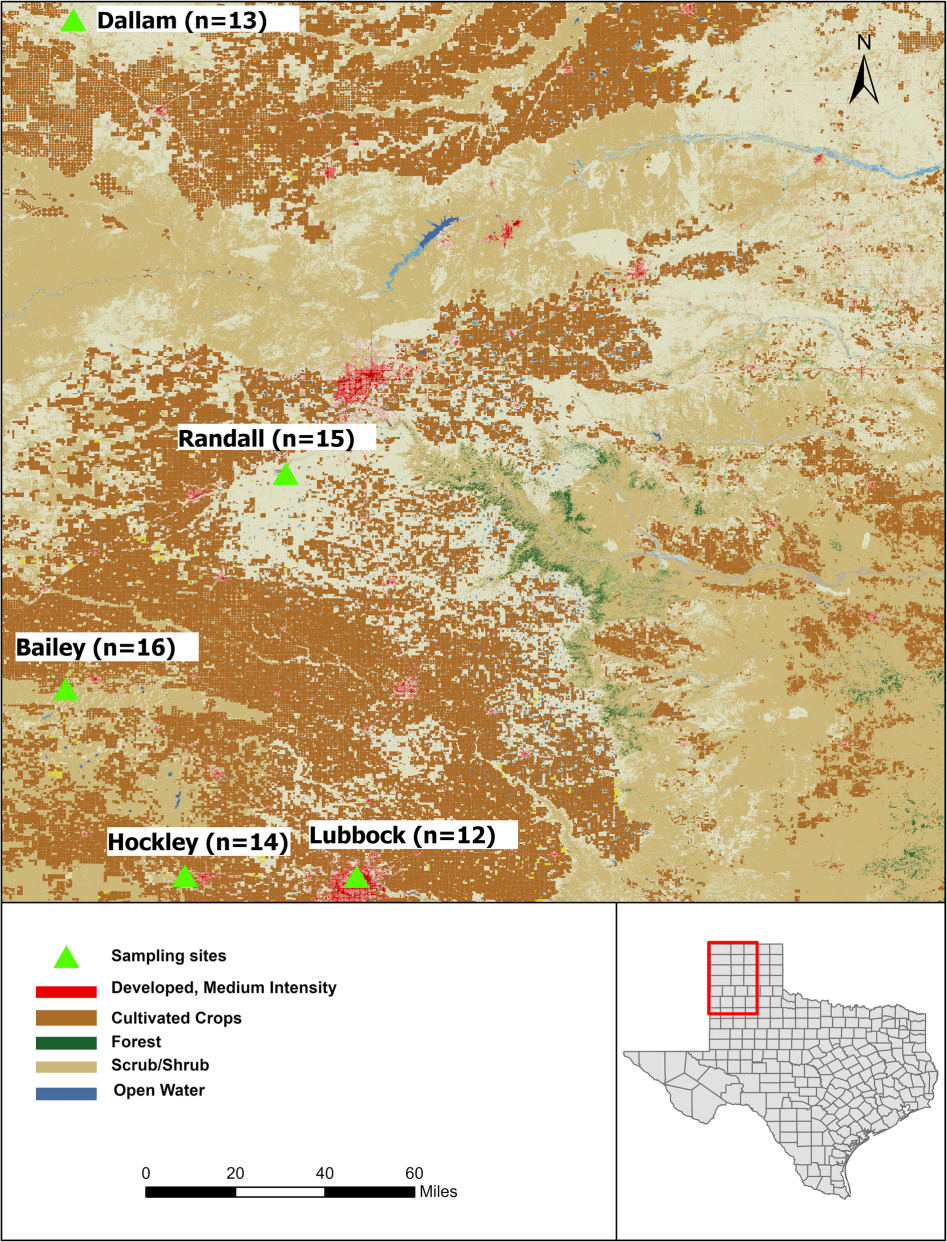
Locations of five sampling sites of *Cynomys ludovicianus* in the Texas Panhandle where ‘n’ is the number of samples drawn from each site. Inset demonstrates the map of Texas.The map was created with ArcGIS Pro (v 2.9.0)

Fecal sampling was conducted at five different Counties in the Texas Panhandle to take into consideration of habitat variation and urbanization (Fig. 8). Lubbock, Hockley, and Bailey were classified as urban habitats for *Cynomys ludovicianus* as these sites are fragmented and surrounded by parks and residential areas which are managed and largely utilized by the public and near to the built-up area of the city (Table 3). In contrast, Randall and Dallam were categorized as rural areas that preserve short-grass prairie habitat; sampling sites were selected from tens to several hundred miles away from urban areas, preventing direct human exposure. Samples were collected during the summer from June to August 2021. Geographic coordinates of sampled sites were determined by handheld GPS (Garmin E10). Bioclimatic data of each sampled site was collected from the national climatic data center (The West Texas Mesonet, https://www.depts.ttu.edu/nwi/research/facilities/wtm/index.php). We used monthly precipitation by summing the daily precipitation at each location to estimate the cumulative precipitation during summer. The average minimum and average maximum temperatures for summer were also determined by using the daily minimum and maximum temperatures. Non-invasive sampling was used to detect bacterial communities in the black-tailed prairie dog population. Fresh fecal samples were collected immediately upon defecation from black-tailed prairie dog colonies. We aimed at collecting fresh fecal pellets (light green in color) early in the morning around active burrows. Sampled burrows were distributed throughout the area of each colony. To increase the rate of the finding of fecal pellets, we applied cluster sampling [96]. When we found a fecal sample, the other member of the group searched the colony area within a radius of approximately 7–8 m to see if further prairie dog feces could be found in that radius [97]. Fresh fecal droppings were drawn using sterile tweezers and transferred in 5 ml sterile transport tubes in Ziploc plastic bags. Each fecal sample was cleaned to remove soil bacteria and stored frozen instantly on dry ice to prevent cross-contamination. Genomic DNA was extracted within 24–48 h of sample collection. The sampling protocol was approved by the Texas Tech University and Institutional Animal Care and Use Committee (IACUC permit #: X21040).

**Table 3:** Sample information including bioclimatic data

Sampling sites	Habitat	Elevation (m)	Cumulative precipitation (cm)	Average minimum temperature (°F)	Average maximum temperature (°F)
Lubbock (*n* = 12)	Urban	970	19.1	63.6	91.5
Hockley (*n* = 14)	Urban	1010	19.8	62.8	91.2
Bailey (*n* = 16)	Urban	1163	18.4	61.4	90.3
Randall (*n* = 15)	Rural	1107	20.1	62.7	89.9
Dallam (*n* = 13)	Rural	1320	22.2	59.9	88.5

### DNA isolation, library preparation, and 16S rRNA amplicon sequencing

DNA was isolated from fresh feces (≤ 150 mg) using Quick-DNA Fecal/Soil Microbe Miniprep Kit (Zymo Research, Irvine, CA, USA) based on the manufacturer’s protocol. The extracted DNA was assessed through electrophoresis in 2% agarose gels and quality and quantity were estimated via spectrophotometry (Nanodrop). The 16S rRNA gene in fecal samples of black-tailed prairie dogs' DNA extracts was PCR amplified with universal primers targeting the V1-V3 regions to amplify ~ 500 bp. The forward primer was designed using Illumina i5 overhang adapter (5´-AATGATACGGCGACCACCGAGATCTACAC-3´), and 28F (5´-GAGTTTGATCNTGGCTCAG-3´) while for the reverse primer, Illumina i7 overhang adapter (5´-CAAGCAGAAGACGGCATACGAGAT-3´) and 519R (5′ GTNTTACNGCGGCKGCTG-3′) [98] were used. The PCR reaction was performed using 3.0 μl of DNA, 1 μl of each 5 μM forward and reverse primers, 12.5 μl repliQa HiFi ToughMix (Quantabio, Beverly, MA, USA), and 7.5 μl PCR Grade Water. For negative control in PCR, we used DNase free water. The PCR amplification was carried out in an Eppendorf Mastercycler (Eppendorf North America, Inc. One Cantiague Road, Westbury, NY) using the following program: 95 °C for 3 min, followed by 35 cycles of 98 °C for 10 s; 54 °C for 40 s; 72 °C for 30 s; 72 °C for 5 min and a final hold at 4 °C. After initial amplification, reactions were purified using 10 nM Tris pH 8.5, AMPure XP beads (NEBNext® sample purification beads, USA), and 80% ethanol on a magnetic stand. In a subsequent PCR, dual index tags were annexed using the Nextera XT Index Kit (S502-S503, S505-S508, S510-S511, and N701-N707, N710-N712) in order to differentiate multiple samples in a single run based on the manufacturer’s protocol (Illumina, San Diego, CA, USA). The settings for index PCR were at 95 °C for 3 min followed by 8 cycles of 95 °C for 30 s, 55 °C for 30 s, 72 °C for 30 s, 72 °C for 5 min with a final step of 4 °C. Following PCR, reactions were cleaned using the AMPure XP beads (NEBNext® sample purification beads, USA). Library quality was measured via Qubit 2.0 Fluorometer (Thermo Scientific, USA) and library quantity was estimated with Bioanalyzer 2100 system (Agilent Technologies, Santa Clara, CA). Libraries were pooled equimolar and appropriately normalized using 10 nM Tris pH 8.5 before sequencing. Pooled libraries were quantified again with Qubit 2.0 Fluorometer (Thermo Scientific, USA) and sequenced for paired 300 bp reads at 10 pM using Illumina MiSeq reagent kit V3 (Illumina, Inc. San Diego, CA, USA) at Texas Tech Genomic Core Facility.

### Sequence processing and taxonomic assignment

Forward and reverse raw reads from Illumina MiSeq were merged using the read stitching algorithm PEAR [99], which requires a minimum overlap of the amplicon to create a consensus sequence. The resulting stitched sequences were then denoised by quality filtering and removed low-quality sequences (Q < 30) and sequences with erroneous base calls. Reads were discarded with more than one error and resulting filtered and stitched sequences were combined into a single fasta file. Sequences were clustered into ZOTUs (zero-radius operational taxonomic units) based on a 100% similarity threshold using USERCH [100] with UNOISE algorithm that compares the similarity and abundances of the sequence to detect whether the sequences are unique bacterial lineages or likely the result of sequence errors. Chimeric sequences were discarded, and the remaining OTU sequences were aligned against version 123 of the SILVA database [101] for a species-level taxonomic assignment using SSU-ALIGN [102]. The chimera-checking algorithm did not allow us to assign sequences of the 16S rRNA gene to phyla level. Phyla were assigned based on BLAST searches against the SILVA database [103]. We generated phylogenetic estimation from the aligned sequences to summarize the evolutionary relationship among ZOTUs using FastTree and rooted the tree at the midpoint [104]. The resulting community matrix (OTU table) was used in which rows were represented by samples, OTUs as columns as well as the number of reads per OTU in each sample as cells for downstream analyses.

### Statistical analyses

The OTU and taxonomy table were imported in R 4.1.2 [105] and analyzed using phyloseq [106], vegan [107], phytools [108], pheatmap [109], reshape2 [110], scales [111], picante [112], ANCOMBC [113], lme4 [114], nlme [115], and AICcmodavg [116]. Data were visualized in ggplot2 [117]. To evaluate the variation in library size across the dataset and to assess if sequencing depth is enough to detect and characterize microbial communities, the rarefaction curve was used to normalize the number of reads by randomly sub-sampled 40,000 reads with a step size of 250. As a result of a low number of sequences, seven of our 77 prior samples were discarded, thus our final dataset consisted of 70 samples. The rarefied dataset was used for further diversity analyses. Rank abundance and incidence abundance plots were used to test the sample divergence in their distribution across the dataset.

### Alpha diversity analyses

Sequencing effort was measured using rarefaction curves of the number of OTU in the samples. Microbiome data were analyzed at the family, genera, and species levels. Linear regression was used using ‘lm’ function to evaluate the relationship between observed OTU richness and the number of reads in the samples which was followed by the Welch Two Sample t-test. We estimated three different alpha diversity metrics to assess the diversity within sampled localities: observed richness (total number of OTUs in a sample), Shannon diversity index (richness and evenness of OTUs in a sample) [118], and Faith’s phylogenetic diversity (phylogenetic distance as a measure of branch length between the observed bacterial species) [119] using ‘estimate_richness’ function. We tested whether alpha diversity indices differed across sampling sites and habitats using ANOVA (analysis of variance). Tukey's HSD test was used to determine the pairwise comparisons between groups. To evaluate the relationship between alpha diversity (observed richness), sampling sites, and habitats, we used a linear regression model. We also performed multiple linear regression to determine whether fecal microbial alpha diversity was confounded by habitat or any significant association between diversity and predictor variables.

### Beta diversity analyses

Compositional differences across host populations were assessed through taxonomic (Bray–Curtis dissimilarity) [120], and phylogenetic metrics (weighted and unweighted UniFrac distance) [121]. To test the predictors associated with community dissimilarity and between-sample variation in multivariate space, we used principal coordinate analysis (PCoA) with ‘capscale’ function based on three beta diversity metrics. The ordination techniques for the microbiome dataset have been recommended by Gloor et al. 2017 [122]. Analyzing the effect of sampling sites and habitat on microbiome community structure was done using permutational analysis of variance (ADONIS) with an ‘adonis’ function on the resulting distance matrices [123]. A significant test result for sampling sites led to pairwise comparisons between sampling sites using post hoc adonis. To further test for differences across sampling sites, we performed multivariate homogeneity of group dispersions based on the distance of each individual relative to the group centroid with ‘betadisper’ function resulting from principal coordinate analysis, and differences in group dispersion were later corroborated by ANOVA. To test which taxa influenced changes in microbiome composition across host populations, we used a stacked bar plot and heatmap for hierarchical clustering based on the mean relative abundance of the family and genera.

### Differential abundance analyses

Following binning OTUs into species level, we conducted differential abundance tests [124]. For species-level differential abundance explained by the variable of interest, we used analysis of compositions of microbiomes with bias correction (ANCOMBC), a statistical approach that accounts for sampling fraction, normalizes the read counts by a process analogous to log-ratio transformations as well as controls the false discovery rates and increasing power. Raw species counts were used as input to ‘ANCOMBC’ function and adjusted *p*-values set to Benjamini-Hochberg (BH) with the rest of the parameters left as defaults. ANCOMBC was implemented to search for the taxa with significantly different relative abundance across sampling sites and habitats and the results were visualized with box plots.

### Random forest classifier

To determine how important differentially expressed bacterial taxa were to the microbial community, a random forest classifier was implemented using the R package ‘randomForest’, an ensemble learning method for classification and regression [125]. A random forest model (RF) based on the habitats with the relative abundance of 220 bacterial species was used as input. RF uses 500 trees and approximately two-thirds of the samples from the original dataset were used to train by random sampling with replacement, whereas one-third of the samples were used to evaluate the accuracy of the tree using ‘out of bag’ (OOB) estimate of the error rate to make it robust against overfitting [126]. Based on the increase in error rate and the number of times splitters are used, an importance score, mean decrease accuracy (MDA), is assigned to each input. It has been demonstrated that RF outperforms support vector machines when analyzing the microbiome data [127, 128].

### Spatial autocorrelation analyses

Haversine distances were used to calculate the distance matrix between the sampling sites. Moran's I [129] test was implemented as part of the ape package [130] for determining the relationship of spatial variables in the form of geographic distance and microbial alpha diversity. To determine whether beta diversity is spatially autocorrelated, we tested the association between community dissimilarity matrices as summarized in Bray–Curtis, weighted and unweighted UniFrac distance matrices and geographic distance matrices using the mantel test [131] as implemented in the vegan package.

### Relationships between environmental factors and microbial diversity

We evaluated the relationship between environmental variables and alpha and beta diversity using Pearson’s correlation coefficient. The linear mixed effect models with corrected Akaike’s Information Criterion (AICc) were used to test the predictability of environmental factors (1) cumulative precipitation (2) elevation (3) average maximum temperature and (4) average minimum temperature on fecal microbial alpha and beta diversity with habitat as a random effect in the models.

## Supplementary Information

**Additional file 1: Table S1.** Summary of Illumina 16S rRNA sequencing data of *Cynomys ludovicianus* fecal microbiome across study sites. **Table S2.** Effect of host locations on the microbiome community composition based on the results of overall and pairwise ADONIS (permutational multivariate analysis of variance using distance matrices) tests. **Table S3.** Alpha (Shannon diversity index) and beta diversity (PCoA 1) models based on AICc model selection. **Table S4.** Comparison of sampling method and gut microbiome composition of black-tailed prairie dogs’ study in North American Great Plains. **Fig. S1.** Rarefaction curves showing species richness as a function of normalized sequence depth for each sample clustered by sites. **Fig. S2.** Species abundance distribution and detection rate across sample datasets (A) Rank abundance plot depicts high-ranking species having higher abundances compared to low-ranking species, and (B) Incidence abundance plot shows about 100 bacterial species being detected in 70% of fecal samples across the dataset. **Fig. S3.** Mean decrease in accuracy (MDA) measures the variable importance of the top 30 bacterial taxa in the Random Forest (RF) model between rural and urban areas with an out-of-bag error rate (11.43%). The taxa are ranked in decreasing order from top to the bottom and the length of the bars corresponds to the degree to which the selected features are important for classification.

## Data Availability

Raw sequence data and meta data can be obtained from the NCBI Sequence Read Archive (SRA) under the BioProject PRJNA869084 (Reviewer Link: https://www.ncbi.nlm.nih.gov/bioproject/PRJNA869084). There was no custom code used, and the main text explains all analyses.
