# Supplementary material for: Fine-scale spatial variation shape fecal microbiome diversity and composition in black-tailed prairie dogs (Cynomys ludovicianus)
Source: BMC Microbiol. 2023 Mar 2;23:51. doi: 10.1186/s12866-023-02778-0 (PMC9979494; doi:10.1186/s12866-023-02778-0)
Supplement: Supplementary file 1 — Additional file 1: Table S1. Summary of Illumina 16S rRNA sequencing data of Cynomys ludovicianus fecal microbiome across study sites. Table S2. Effect of host locations on the microbiome community composition based on the results of overall and pairwise ADONIS (permutational multivariate analysis of variance using distance matrices) tests. Table S3. Alpha (Shannon diversity index) and beta diversity (PCoA 1) models based on AICc model selection. Table S4. Comparison of sampling method and gut microbiome composition of black-tailed prairie dogs’ study in North American Great Plains. Fig. S1. Rarefaction curves showing species richness as a function of normalized sequence depth for each sample clustered by sites. Fig. S2. Species abundance distribution and detection rate across sample datasets (A) Rank abundance plot depicts high-ranking species having higher abundances compared to low-ranking species, and (B) Incidence abundance plot shows about 100 bacterial species being detected in 70% of fecal samples across the dataset. Fig. S3. Mean decrease in accuracy (MDA) measures the variable importance of the top 30 bacterial taxa in the Random Forest (RF) model between rural and urban areas with an out-of-bag error rate (11.43%). The taxa are ranked in decreasing order from top to the bottom and the length of the bars corresponds to the degree to which the selected features are important for classification. [file 12866_2023_2778_MOESM1_ESM.docx]

**Supplementary Data**

Table S1 Summary of Illumina 16S rRNA sequencing data of *Cynomys ludovicianus* fecal microbiome across study sites

| Sites | Sample ID | Reads | Filtered reads | Observed OTUs | Shannon | Faith’s PD |
| --- | --- | --- | --- | --- | --- | --- |
| Lubbock | BH22621 | 77295 | 54964 | 1733 | 6.84 | 13.14 |
|  | BH42621 | 91316 | 65179 | 1678 | 6.77 | 12.73 |
|  | BH152521 | 83497 | 59364 | 1729 | 6.77 | 12.86 |
|  | BH252521 | 74216 | 54097 | 1916 | 6.94 | 13.30 |
|  | MP18111 | 51568 | 37568 | 647 | 5.17 | 8.96 |
|  | MP18112 | 55870 | 42509 | 1168 | 6.25 | 10.89 |
|  | MP18113 | 56935 | 41591 | 1094 | 6.16 | 10.16 |
|  | MP18114 | 65203 | 50207 | 1922 | 6.93 | 14.09 |
|  | MP18115 | 105295 | 81544 | 1773 | 6.77 | 12.51 |
|  | MP18116 | 89500 | 69002 | 1023 | 6.02 | 9.67 |
|  | ID352521 | 40676 | 28950 | 1482 | 6.59 | 11.28 |
|  | ID452521 | 48011 | 32234 | 1608 | 6.71 | 11.89 |
| Randall | BL641 | 72630 | 52130 | 1679 | 6.68 | 11.93 |
|  | BL642 | 56565 | 40933 | 1528 | 6.52 | 11.39 |
|  | BL643 | 73853 | 53193 | 1708 | 6.74 | 11.62 |
|  | BL644 | 66696 | 46337 | 1705 | 6.68 | 11.80 |
|  | BL645 | 75130 | 52812 | 1901 | 6.95 | 12.94 |
|  | BL646 | 60496 | 41036 | 1771 | 6.75 | 12.31 |
|  | BL647 | 92870 | 65413 | 1910 | 6.99 | 12.98 |
|  | BL648 | 88695 | 63855 | 1733 | 6.77 | 11.84 |
|  | BL649 | 71601 | 52182 | 1829 | 6.54 | 12.92 |
|  | BL6410 | 70439 | 52676 | 1751 | 6.35 | 11.93 |
|  | BL6411 | 97095 | 68788 | 1233 | 6.27 | 9.58 |
|  | BL6412 | 49993 | 36002 | 1698 | 6.71 | 11.88 |
|  | BL6413 | 150685 | 109441 | 1331 | 5.48 | 11.18 |
|  | BL6414 | 87710 | 62767 | 1808 | 6.77 | 13.27 |
|  | BL6415 | 90038 | 64745 | 1586 | 6.62 | 11.11 |
| Hockley | LV641 | 36755 | 26828 | 1077 | 5.98 | 9.88 |
|  | LV642 | 81899 | 60198 | 1205 | 6.07 | 9.91 |
|  | LV644 | 33270 | 23824 | 1189 | 6.22 | 10.53 |
|  | LV645 | 43535 | 30595 | 1307 | 6.22 | 10.78 |
|  | SM641 | 74984 | 53411 | 1415 | 6.50 | 11.94 |
|  | SM642 | 70557 | 51159 | 1565 | 6.67 | 11.91 |
|  | SM643 | 105297 | 78120 | 1363 | 6.33 | 10.87 |
|  | SM644 | 95878 | 69991 | 1311 | 6.27 | 11.06 |
|  | SM645 | 81012 | 58160 | 1559 | 6.58 | 12.87 |
|  | SM646 | 98651 | 69497 | 972 | 6.00 | 9.29 |
|  | SM647 | 70155 | 49930 | 1602 | 6.65 | 12.96 |
|  | SM648 | 43622 | 30827 | 1129 | 6.17 | 10.04 |
|  | SM649 | 81545 | 58333 | 1225 | 6.19 | 10.52 |
|  | SMI641 | 92127 | 66357 | 1585 | 6.69 | 10.82 |
| Bailey | M6121 | 77937 | 57458 | 1250 | 6.35 | 11.09 |
|  | M6122 | 50915 | 32855 | 1130 | 6.21 | 10.59 |
|  | M6123 | 109928 | 81582 | 1325 | 6.21 | 10.93 |
|  | M6124 | 28178 | 15839 | 1053 | 6.17 | 10.52 |
|  | M6125 | 66589 | 47998 | 1199 | 6.30 | 10.79 |
|  | M6126 | 58992 | 43434 | 1198 | 6.29 | 10.56 |
|  | M6127 | 357624 | 256779 | 1614 | 6.35 | 12.98 |
|  | M6128 | 127413 | 104072 | 1718 | 6.66 | 12.38 |
|  | M6129 | 30203 | 23528 | 1252 | 6.22 | 10.73 |
|  | M61210 | 78538 | 58841 | 1281 | 6.20 | 10.37 |
|  | N6121 | 124427 | 97636 | 1323 | 6.31 | 11.39 |
|  | N6122 | 70638 | 56080 | 1258 | 5.99 | 10.26 |
|  | N6123 | 72956 | 56789 | 1093 | 6.17 | 10.23 |
|  | N6124 | 74422 | 55543 | 1114 | 6.08 | 10.33 |
|  | N6125 | 69107 | 47439 | 1039 | 6.12 | 10.55 |
|  | N6126 | 75448 | 53711 | 1067 | 5.72 | 10.15 |
| Dallam | RB681 | 67526 | 48401 | 1677 | 6.73 | 12.38 |
|  | RB682 | 62888 | 46430 | 1552 | 6.65 | 11.91 |
|  | RB683 | 106628 | 78895 | 1819 | 6.81 | 13.62 |
|  | RB684 | 64624 | 47353 | 1642 | 6.74 | 12.04 |
|  | RB685 | 54201 | 37337 | 1165 | 6.05 | 9.61 |
|  | RB686 | 79835 | 58307 | 1847 | 6.93 | 12.66 |
|  | RB687 | 71679 | 49086 | 1828 | 6.96 | 13.26 |
|  | RB688 | 73365 | 53881 | 1603 | 6.71 | 12.21 |
|  | RB689 | 88499 | 65052 | 1878 | 6.91 | 12.32 |
|  | RB6811 | 35826 | 27208 | 1790 | 6.90 | 12.69 |
|  | RB6812 | 53133 | 40000 | 1796 | 6.84 | 13.18 |
|  | RB6813 | 49734 | 37062 | 1509 | 6.17 | 12.10 |
|  | RB6814 | 73242 | 54602 | 1733 | 6.77 | 12.82 |
| Total | 70 | 5,407,660 | 3,939,947 | 5,118 |  |  |

Table S2 Effect of host locations on the microbiome community composition based on the results of overall and pairwise ADONIS (permutational multivariate analysis of variance using distance matrices) tests

| Comparison | Metrics | F-statistic | R^2^ | p-value |
| --- | --- | --- | --- | --- |
| Overall | Bray-Curtis | 3.49 | 0.17 | <0.01 |
|  | Unweighted Unifrac | 4.33 | 0.21 | <0.01 |
|  | Weighted Unifrac | 3.54 | 0.18 | <0.01 |
| Bailey/Dallam | Bray-Curtis | 3.20 | 0.14 | <0.01 |
|  | Unweighted Unifrac | 4.53 | 0.19 | <0.01 |
|  | Weighted Unifrac | 2.69 | 0.12 | <0.01 |
| Bailey/Hockley | Bray-Curtis | 3.29 | 0.15 | <0.01 |
|  | Unweighted Unifrac | 4.81 | 0.21 | <0.01 |
|  | Weighted Unifrac | 3.32 | 0.15 | <0.01 |
| Bailey/Lubbock | Bray-Curtis | 4.07 | 0.17 | <0.01 |
|  | Unweighted Unifrac | 6.04 | 0.23 | <0.01 |
|  | Weighted Unifrac | 3.64 | 0.16 | <0.01 |
| Bailey/Randall | Bray-Curtis | 2.75 | 0.13 | <0.01 |
|  | Unweighted Unifrac | 3.89 | 0.17 | <0.01 |
|  | Weighted Unifrac | 2.57 | 0.12 | 0.01 |
| Dallam/Hockley | Bray-Curtis | 3.35 | 0.14 | <0.01 |
|  | Unweighted Unifrac | 5.42 | 0.21 | <0.01 |
|  | Weighted Unifrac | 2.96 | 0.12 | 0.01 |
| Dallam/Lubbock | Bray-Curtis | 3.78 | 0.15 | <0.01 |
|  | Unweighted Unifrac | 5.57 | 0.22 | <0.01 |
|  | Weighted Unifrac | 3.48 | 0.14 | <0.01 |
| Dallam/Randall | Bray-Curtis | 2.61 | 0.11 | 0.02 |
|  | Unweighted Unifrac | 3.35 | 0.14 | <0.01 |
|  | Weighted Unifrac | 2.61 | 0.12 | <0.01 |
| Hockley/Lubbock | Bray-Curtis | 4.50 | 0.18 | <0.01 |
|  | Unweighted Unifrac | 7.78 | 0.27 | <0.01 |
|  | Weighted Unifrac | 5.16 | 0.20 | <0.01 |
| Hockley/Randall | Bray-Curtis | 3.35 | 0.15 | <0.01 |
|  | Unweighted Unifrac | 5.43 | 0.22 | <0.01 |
|  | Weighted Unifrac | 3.79 | 0.16 | <0.01 |
| Lubbock/Randall | Bray-Curtis | 3.81 | 0.16 | <0.01 |
|  | Unweighted Unifrac | 5.75 | 0.22 | <0.01 |
|  | Weighted Unifrac | 4.11 | 0.17 | <0.01 |

Table S3 Alpha (Shannon diversity index) and beta diversity (PCoA 1) models based on AICc model selection

| \| Alpha diversity models \| K \| AICc \| ΔAIC_c_ \| W_i_ \| Cumulative W_i_ \| LL \| \| --- \| --- \| --- \| --- \| --- \| --- \| --- \| \| Average maximum temperature \| 3 \| 47.50 \| 0.00 \| 0.39 \| 0.39 \| -20.57 \| \| Average maximum temperature + Average minimum temperature \| 4 \| 48.03 \| 0.52 \| 0.30 \| 0.69 \| -19.71 \| \| Cumulative precipitation + Average maximum temperature \| 4 \| 49.61 \| 2.10 \| 0.14 \| 0.82 \| -20.50 \| \| Cumulative precipitation + Average maximum temperature + Average minimum temperature \| 5 \| 50.33 \| 2.82 \| 0.09 \| 0.92 \| -19.70 \| \| Cumulative precipitation \| 3 \| 51.36 \| 3.86 \| 0.06 \| 0.97 \| -22.50 \| \| Cumulative precipitation + Average minimum temperature \| 4 \| 53.41 \| 5.90 \| 0.02 \| 0.99 \| -22.40 \| \| Average minimum temperature \| 4 \| 55.78 \| 8.28 \| 0.01 \| 1.00 \| -24.71 \| \| Beta diversity models \|  \|  \|  \|  \|  \|  \| \| Average maximum temperature \| 3 \| -158.90 \| 0.00 \| 0.37 \| 0.37 \| 83.26 \| \| Average maximum temperature + Average minimum temperature \| 4 \| -157.32 \| 0.00 \| 0.06 \| 0.91 \| 83.34 \| \| Elevation + Average maximum temperature + Average minimum temperature \| 5 \| -156.82 \| 1.08 \| 0.22 \| 0.59 \| 83.88 \| \| Cumulative precipitation + Elevation + Average maximum temperature + Average minimum temperature \| 6 \| -155.94 \| 1.96 \| 0.14 \| 0.73 \| 84.63 \| \| Cumulative precipitation + Average maximum temperature + Average minimum temperature \| 5 \| -155.59 \| 2.31 \| 0.12 \| 0.84 \| 83.26 \| \| Cumulative precipitation + Elevation + Average maximum temperature \| 5 \| -153.65 \| 4.25 \| 0.04 \| 0.95 \| 82.29 \| \| Cumulative precipitation + Average maximum temperature \| 4 \| -152.78 \| 5.12 \| 0.03 \| 0.98 \| 80.70 \| \| Elevation + Average maximum temperature \| 4 \| -152.24 \| 5.66 \| 0.02 \| 1.00 \| 80.43 \| \| Elevation + Cumulative precipitation \| 4 \| -137.35 \| 20.55 \| 0.00 \| 1.00 \| 72.98 \| \| Cumulative precipitation \| 3 \| -136.74 \| 21.16 \| 0.00 \| 1.00 \| 71.55 \| \| Cumulative precipitation + Elevation + Average minimum temperature \| 5 \| -136.03 \| 21.87 \| 0.00 \| 1.00 \| 73.48 \| \| Cumulative precipitation + Average minimum temperature \| 4 \| -134.67 \| 23.23 \| 0.00 \| 1.00 \| 71.64 \| \| Average minimum temperature \| 3 \| -122.29 \| 35.61 \| 0.00 \| 1.00 \| 64.33 \| \| Elevation + Average minimum temperature \| 4 \| -120.36 \| 37.54 \| 0.00 \| 1.00 \| 64.49 \| \| Elevation \| 3 \| -99.22 \| 58.68 \| 0.00 \| 1.00 \| 52.79 \| |
| --- | --- | --- | --- | --- | --- | --- | --- | --- | --- | --- | --- | --- | --- | --- | --- | --- | --- | --- | --- | --- | --- | --- | --- | --- | --- | --- | --- | --- | --- | --- | --- | --- | --- | --- | --- | --- | --- | --- | --- | --- | --- | --- | --- | --- | --- | --- | --- | --- | --- | --- | --- | --- | --- | --- | --- | --- | --- | --- | --- | --- | --- | --- | --- | --- | --- | --- | --- | --- | --- | --- | --- | --- | --- | --- | --- | --- | --- | --- | --- | --- | --- | --- | --- | --- | --- | --- | --- | --- | --- | --- | --- | --- | --- | --- | --- | --- | --- | --- | --- | --- | --- | --- | --- | --- | --- | --- | --- | --- | --- | --- | --- | --- | --- | --- | --- | --- | --- | --- | --- | --- | --- | --- | --- | --- | --- | --- | --- | --- | --- | --- | --- | --- | --- | --- | --- | --- | --- | --- | --- | --- | --- | --- | --- | --- | --- | --- | --- | --- | --- | --- | --- | --- | --- | --- | --- | --- | --- | --- | --- | --- | --- | --- | --- | --- | --- | --- | --- | --- |

Table S4 Comparison of sampling method and gut microbiome composition of black-tailed prairie dogs’ study in North American Great Plains

| Study area | Sample size | Sample type | Variable regions amplified | Phyla identified | % of Firmicutes | % of Bacteroidetes | Source |
| --- | --- | --- | --- | --- | --- | --- | --- |
| Chihuahua, Mexico | n=10 | Fecal | V3-V4 | 12 | 83.1 | 9.9 | Pacheco-Torres et al. 2019 |
| Kansas, USA | n=58 | Fecal and Cecal | V4 | 28 | 75.0 | 1.8 | Rooney et al. 2021 |
| Texas, USA | n=70 | Fecal | V1-V3 | 11 | 39.6 | 57.3 | This study |


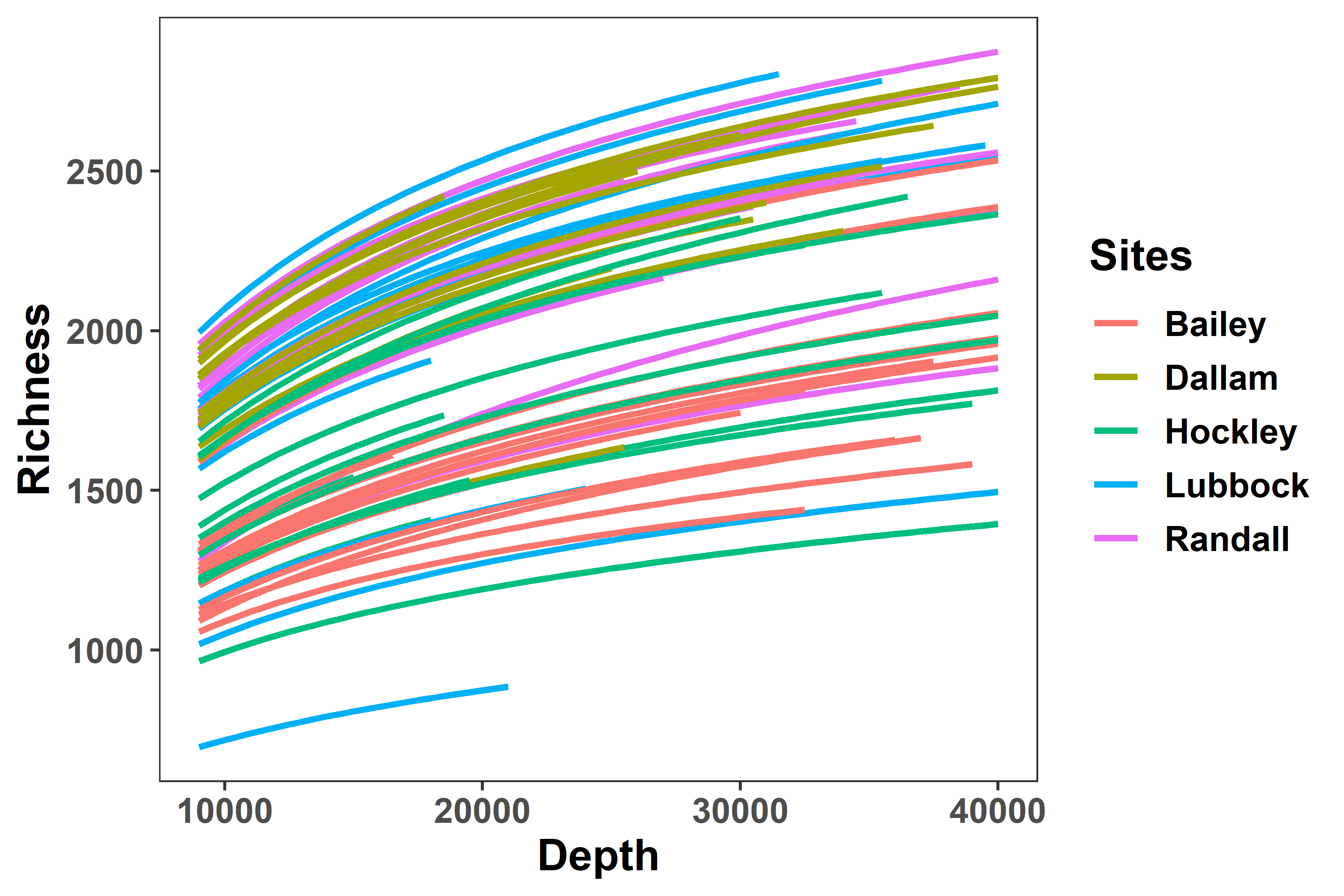


Fig. S1 Rarefaction curves showing species richness as a function of normalized sequence depth for each sample clustered by sites

(A) (B)


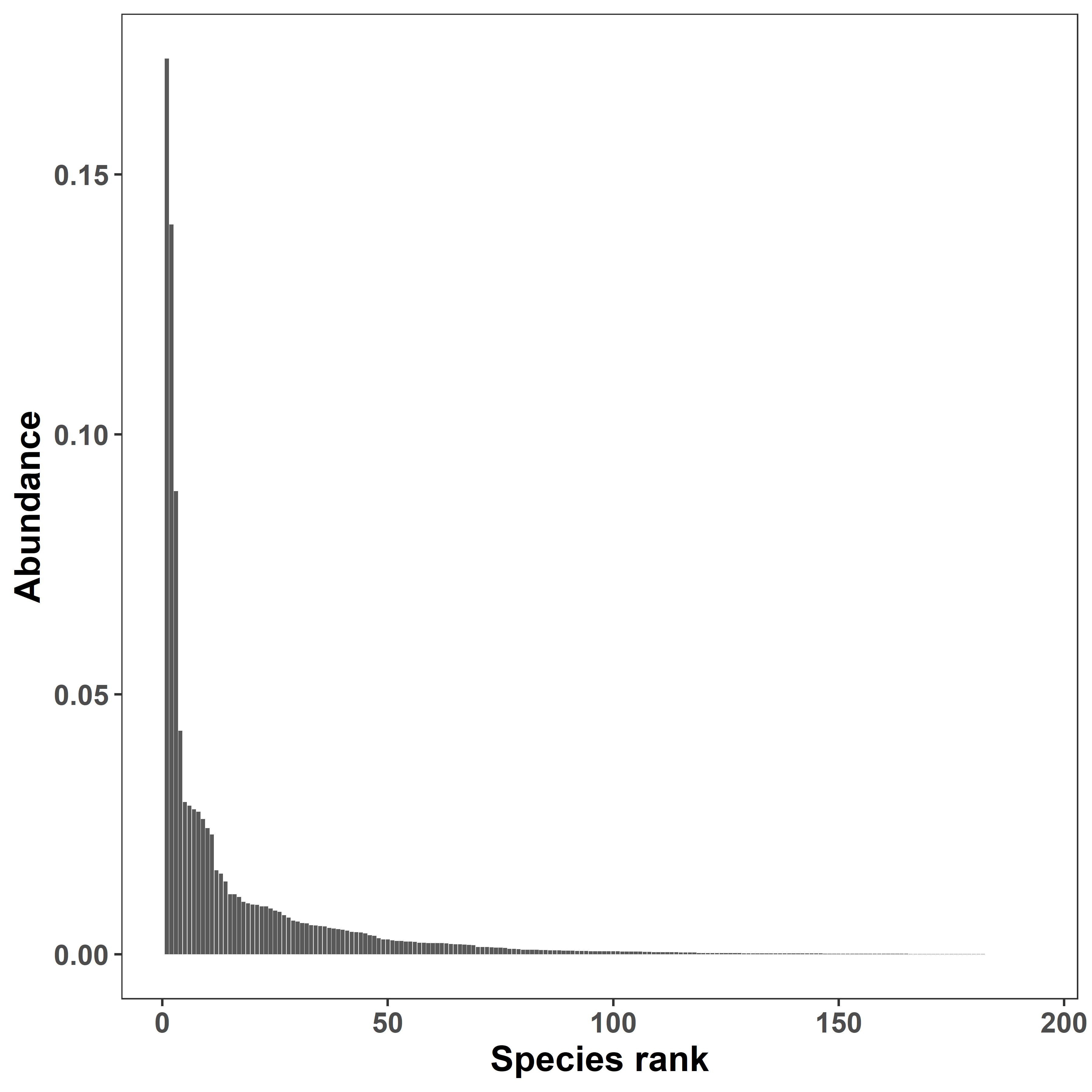

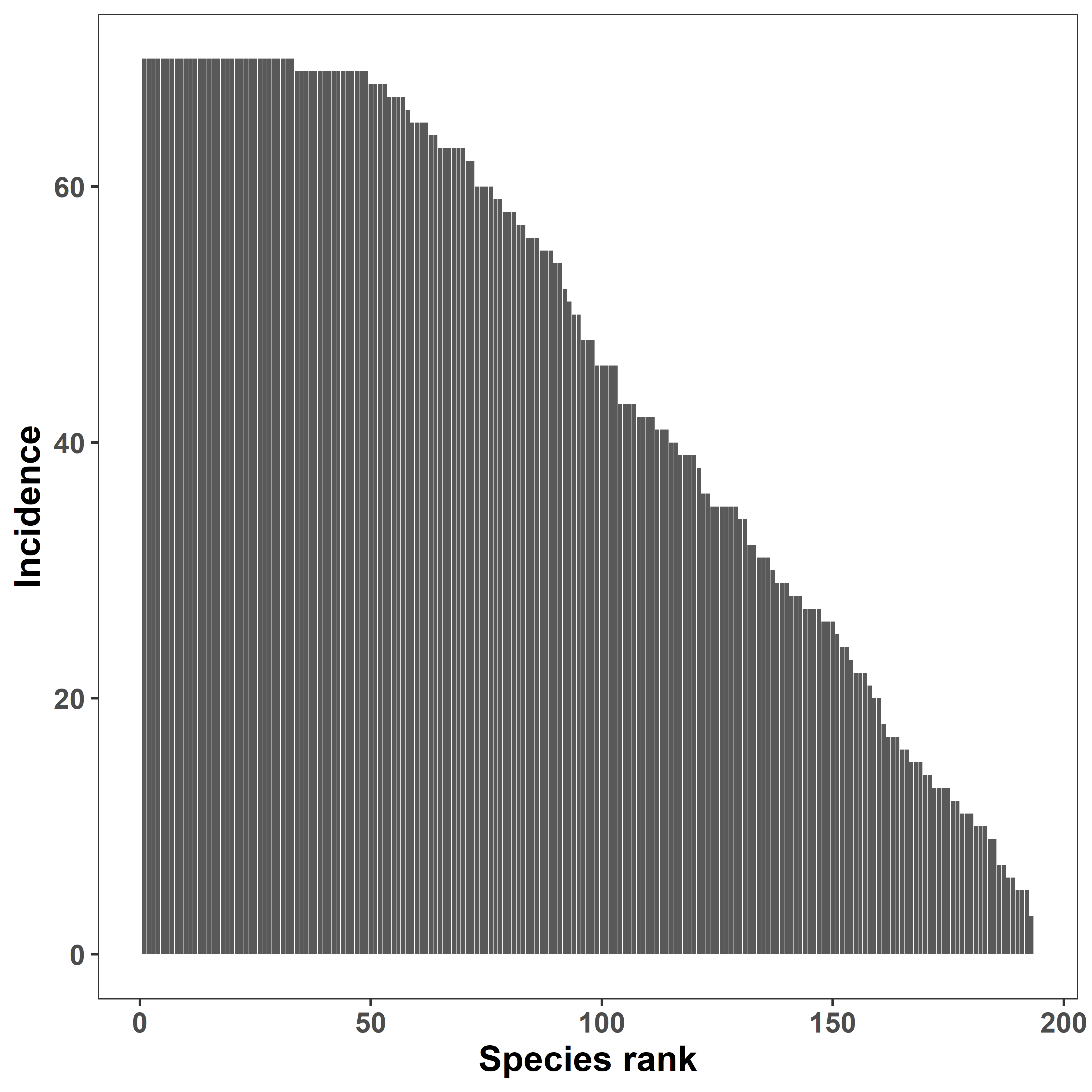


Fig. S2 Species abundance distribution and detection rate across sample datasets (A) Rank abundance plot depicts high-ranking species having higher abundances compared to low-ranking species, and (B) Incidence abundance plot shows about 100 bacterial species being detected in 70% of fecal samples across the dataset


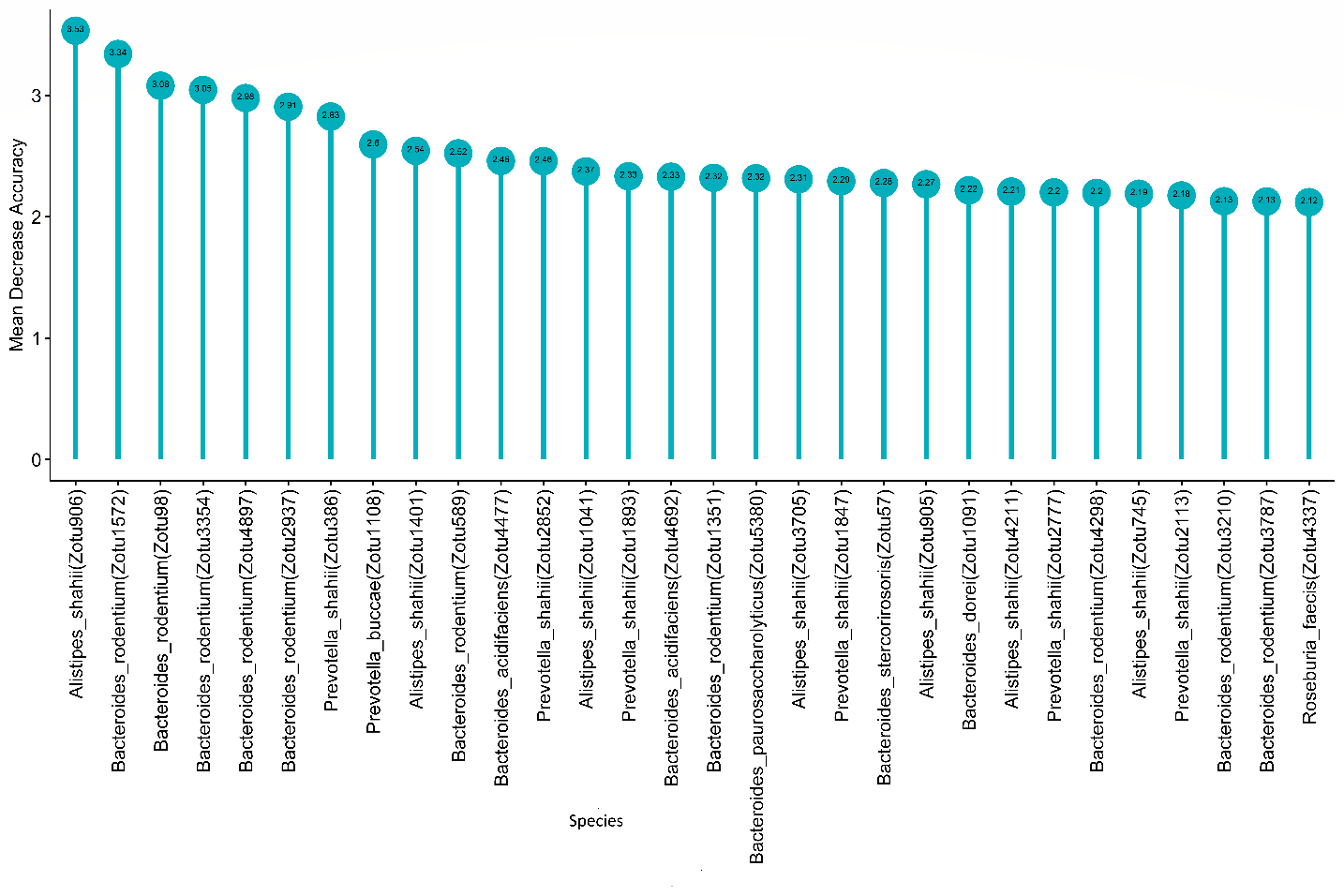


Fig. S3 Mean decrease in accuracy (MDA) measures the variable importance of the top 30 bacterial taxa in the Random Forest (RF) model between rural and urban areas with an out-of-bag error rate (11.43%). The taxa are ranked in decreasing order from top to the bottom and the length of the bars corresponds to the degree to which the selected features are important for classification
